# ROS homeostasis in cell fate, pathophysiology, and therapeutic interventions

**DOI:** 10.1186/s43556-025-00338-8

**Published:** 2025-10-30

**Authors:** Yuan-Shen Chen, Hui-Xiang Tian, Ding-Chao Rong, Luozixian Wang, Shan Chen, Jun Zeng, Heng Xu, Jie Mei, Lei-Yun Wang, Yu-Ligh Liou, Hong-Hao Zhou

**Affiliations:** 1 Key Specialty of Clinical Pharmacy, The First Affiliated Hospital of Guangdong Pharmaceutical University, Guangzhou, 510080 People’s Republic of China; 2 Department of Clinical Pharmacology, Xiangya Hospital, Institute of Clinical Pharmacology, Hunan Key Laboratory of Pharmacogenetics, Central South University, Changsha, Hunan 410008 People’s Republic of China; 3 Tianfu Jincheng Laboratory, Chengdu, Sichuan 610052 People’s Republic of China; 4 Department of Orthopedics, The First Affiliated Hospital of Shaoyang University, Shaoyang, Hunan 422000 People’s Republic of China; 5 Centre for Eye Research Australia, Royal Victorian Eye and Ear Hospital, Melbourne, VIC Australia; 6 Ophthalmology, Department of Surgery, The University of Melbourne, Melbourne, VIC Australia; 7 State Key Laboratory of Oncogenes and Related Genes, Department of Oncology, Shanghai General Hospital, Shanghai Jiao Tong University School of Medicine, Shanghai, 200080 People’s Republic of China; 8 Department of Thoracic Surgery, Xiangya Hospital, Central South University, Changsha, Hunan Province 410008 People’s Republic of China; 9 Department of Laboratory Medicine/Research Center of Clinical Laboratory Medicine, West China Hospital, Sichuan University, Chengdu, Sichuan 610041 People’s Republic of China; 10 Department of Pharmacy, Traditional Chinese and Western Medicine Hospital of Wuhan, Tongji Medical College, Huazhong University of Science and Technology, Hubei Province, Wuhan, 430022 People’s Republic of China

## Abstract

Reactive oxygen species (ROS) homeostasis is an essential process that enables cells dynamically regulate their ROS levels, thereby ensuring survival and the execution of diverse physiological functions. ROS, a group of highly reactive molecules that serve as both critical signaling molecules and potential toxic agents, are central regulators of this process. Dysregulation of ROS homeostasis can impair cellular and organismal physiology, ultimately contributing to disease pathogenesis, which is a phenomenon observed throughout the lifespan. However, the precise mechanisms underlying these processes remain poorly understood, and the therapeutic potential of targeting ROS homeostasis regulation for disease intervention has not been systematically elucidated. This review provides a comprehensive overview of the diverse roles of ROS and their metabolic associations. It offers an in-depth discussion of the regulatory mechanisms underlying ROS homeostasis and their influence on processes such as cellular metabolism, cell death, and cell survival. By modulating cell fate, ROS play a broad and integral role in the pathogenesis of various diseases. Finally, this review systematically summarizes therapeutic interventions targeting ROS homeostasis. By elucidating the critical roles of ROS homeostasis in cellular physiology and disease treatment, this review aims to advance the discovery of potential biomarkers as well as the development of novel therapeutic approaches based on ROS homeostasis.

## Introduction

At the cellular level, cells represent the fundamental functional units of tissues, and their viability and functionality rely heavily on the maintenance of a stable intracellular homeostatic environment throughout tissue development and systemic physiological processes [1]. From the onset of biological development, intracellular reactive oxygen species (ROS) homeostasis is self-regulated to ensure the maintenance of ROS levels and a dynamic equilibrium within cells and tissues [2, 3]. Intracellular ROS homeostasis is characterized by the ability of cells to adapt to changing conditions such as hypoxia, hyperoxia, and oxidative stress [4–6], while disruptions in this equilibrium can have significant implications for cell fate [7], disease progression [8], and therapeutic responses [9].

ROS homeostasis relies on the dynamic equilibrium of intracellular redox reactions [10, 11]. Therefore, processes such as hypoxia and hyperoxia can disrupt oxidative balance and ultimately induce oxidative stress [12, 13], with ROS being one of the main participants [14]. ROS are a collection of oxidative molecules with various biological functions, including the superoxide anion (•O_2_^–^), hydrogen peroxide (H_2_O_2_), and ozone (O_3_) [15]. Different ROS regulate various aspects of cell fate [16], from cell metabolism [17, 18], cell differentiation [19, 20] and cell death [21], thereby affecting the overall fate of tissues and organisms [22]. Indeed, numerous studies have reported the association between disrupted ROS homeostasis and disease progression, including cardiovascular diseases [23], neurodegenerative diseases [24], metabolic diseases [25], reproductive system diseases [26], digestive diseases [27], respiratory system diseases [28], cancers [29], inflammation [30] and infectious diseases [31]. These diseases all involve the critical role of ROS and mediated oxidative stress [32, 33]. Specific mechanisms may involve oxidative damage caused by excessive ROS production, and the persistent accumulation of such damage can lead to further cellular and organismal dysfunction, ultimately inducing various diseases [34]. Given the important role of ROS and the ROS homeostasis in the pathogenesis of diverse diseases, modulating ROS levels and regulating ROS homeostasis has emerged as a promising therapeutic approach [22]. For instance, the development of certain antioxidants has shown promise in mitigating oxidative stress, reducing inflammation, and restoring tissue homeostasis [9]. Additionally, certain pro-oxidative stress regulators have been shown to enhance tumor cells clearance by modulating ROS levels and inducing pathways such as ferroptosis [35]. Other potential therapeutic strategies include oxygen therapy and ROS adaptation regulation, aiming to modulate ROS homeostasis to promote tissue health.

**Fig. 1:**
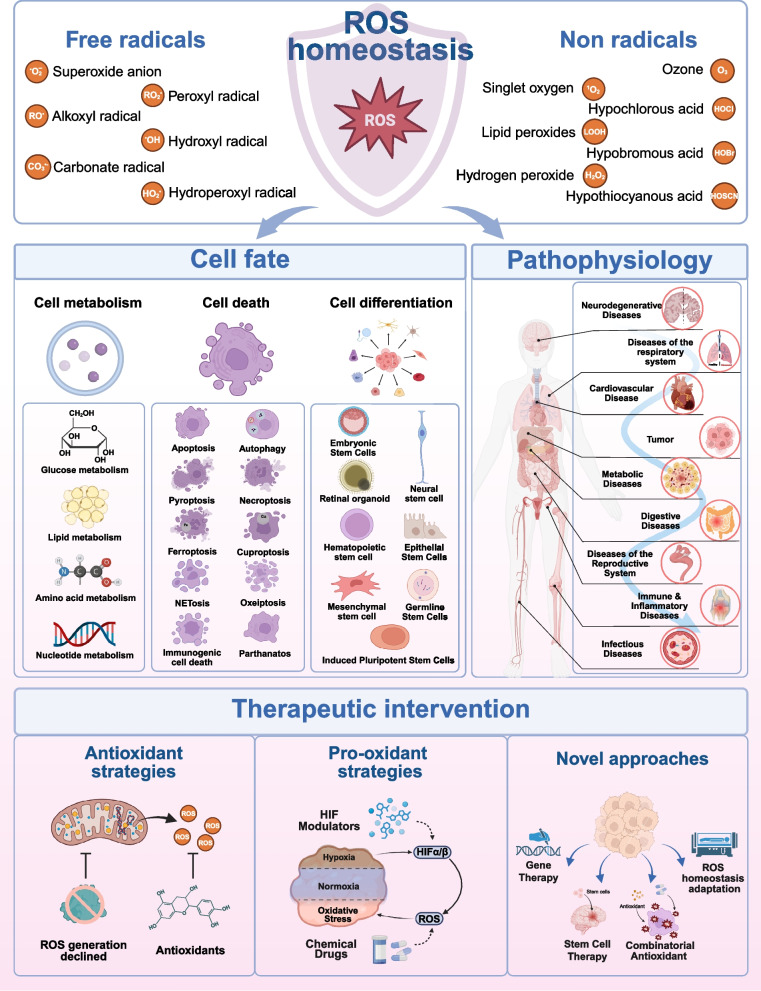
ROS and ROS homeostasis. The coordination between radical and non-radical ROS maintains ROS homeostasis and ultimately regulates cellular fate, including metabolism, survival, death, and stemness maintenance. Conversely, disruption of ROS homeostasis contribute to disease pathogenesis. Therefore, therapeutic strategies targeting ROS homeostasis hold significant implications for modulating the cellular oxidative environment and advancing clinical therapy

In summary, considering the broad impact of ROS homeostasis on cell fate, physiopathology and therapeutic intervention, this review systematically explores the regulatory mechanisms of ROS homeostasis and highlights the pivotal role of the ROS in cell physiology. Given the chronic effects of oxidative homeostasis imbalance and ROS accumulation across diverse diseases, we further discuss their pathological mechanisms and contributions to disease progression and individual life courses. Additionally, to explore the therapeutic potential of ROS homeostasis modulation in disease treatment, this review comprehensively summarizes existing drugs and intervention strategies targeting ROS hemeostasis, while also exploring emerging therapeutic approaches. These insights hold significant value in enhancing our comprehension of disease mechanisms and informing the development of novel therapeutic strategies (Fig. 1).

## Intracellular ROS homeostasis

**Fig. 2:**
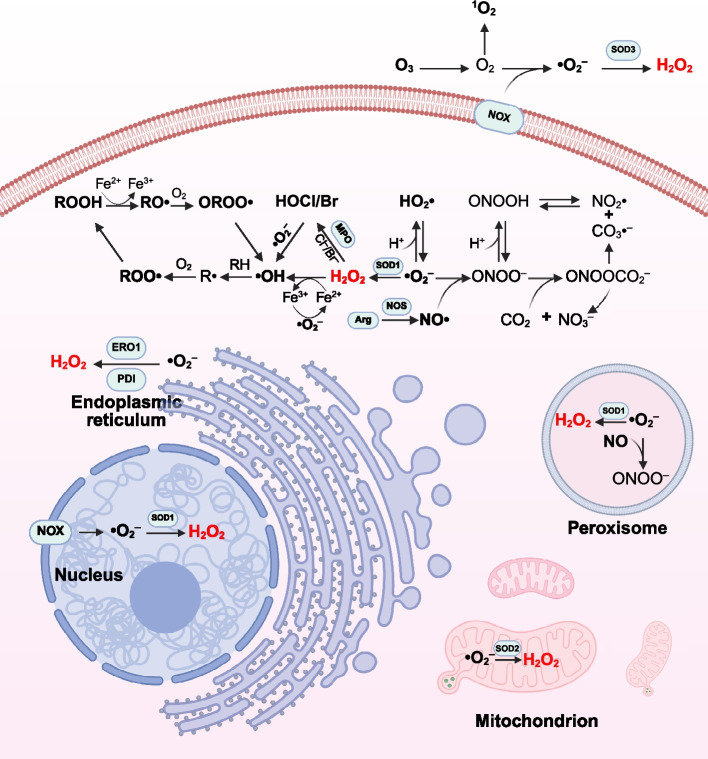
Schematic illustration of ROS generation and metabolism. The production and conversion of different types of ROS mainly occur in the nucleus, mitochondrion, endoplasmic reticulum, peroxisome and cytoplasm, involving free radicals (•O_2_^–^, •OH, HO₂•, RO₂•, RO• and CO₃•⁻) and non-free radicals (H₂O₂, O₃, ^1^O₂, HOCl, HOBr, LOOH and HOSCN)

ROS are a class of oxygen-containing and highly reactive substances, generated within cells from both endogenous and exogenous sources [36]. They primarily include •O_2_^–^, hydroxyl radical (•OH), hydroperoxyl radical (HO₂•), peroxyl radical (RO₂•), alkoxyl radical (RO•), carbonate radical (CO₃•⁻), and other free radicals, as well as non-radicals such as H_2_O_2_, O_3_, singlet oxygen (^1^O₂), hypochlorous acid (HOCl), hypobromous acid (HOBr), lipid peroxides (LOOH), and hypothiocyanous acid (HOSCN) [37–39]. Collectively, these molecules regulate the ROS homeostasis within cells (Fig. 2).

### Categories of intracellular ROS

#### Free radicals

##### Superoxide anion (•O_2_^–^)

•O_2_^–^ is a reactive form of molecular oxygen generated through the one-electron reduction of the oxygen. It is primarily found in plants and animals, playing a significant role in various stages of cellular fate regulation [40, 41]. •O_2_^–^ is the main initial product of ROS, typically produced from electron leakage in the mitochondrial respiratory chain and stored in the mitochondria during cellular diffusion [42–44]. It possesses strong oxidative properties and can react with reductive substances or biomolecules within the cell, thereby regulating redox reactions and influencing the expression of intracellular signaling molecules [45]. For example, superoxide dismutase (SOD) converts •O_2_^–^ into the relatively less reactive H₂O₂ [46]. This conversion can lower intracellular oxidative stress, provided that the H₂O₂ formed is promptly removed by downstream antioxidant enzymes [47], because H₂O₂, although less reactive than •O_2_^–^, is still a ROS. If it accumulates, in the presence of transition metal ions (such as Fe^2+^), H₂O₂ can undergo the Fenton reaction to generate the highly toxic •OH [48]. Moreover, this conversion also promotes cell proliferation and stress responses by regulating various signaling pathways including the NADPH oxidase (NOX) family proteins [37, 49]. Clearly, the oxidative impacts of ROS molecules depend not only on redox potential but also on lifetime and concentration. As such, high amounts of H₂O₂ would lead to more "oxidative stress" than similar concentrations of •O_2_^–^. Additionally, •O_2_^–^ can react with nitric oxide (NO) to generate excessive ONOO⁻, activating caspases, causing lipid peroxidation, and leading to either apoptosis or necrosis [49].

##### Hydroperoxyl radicals (HO₂•)

HO₂•, a form of ROS, forms as an intermediate radical through the protonation of •O_2_^–^ [50]. This radical exists in dynamic equilibrium with •O₂⁻. Due to their high reactivity, HO₂• can react with various biomolecules and are important participants in oxidative stress processes [51]. Intracellularly, HO₂• is primarily localized in regions experiencing severe oxidative stress, such as mitochondria, peroxisomes, and near the cell membrane. The role of HO₂• in oxidative stress and cellular signaling is closely related to the progression of various diseases, including Alzheimer's disease and atherosclerosis [52, 53].

##### Hydroxyl radical (•OH)

•OH is an extremely reactive radical primarily generated in plant and animal cells through the Fenton and Haber–Weiss reactions, especially in iron- and oxygen-rich areas such as mitochondria and lysosomes [54]. Its high reactivity limits its diffusion, causing damage to macromolecules like cell membranes, proteins, and DNA [55, 56]. For instance, •OH can oxidize DNA bases, leading to DNA strand breaks that may result in mutations or tumorigenesis [57, 58]. Moreover, it can initiate lipid peroxidation [59], disrupting cell membrane integrity and impairing cellular signaling and membrane function [60]. Such damage can further lead to mitochondrial dysfunction, triggering apoptosis or necroptosis, which represents a key pathological process in oxidative stress [61].

##### Peroxyl radicals (RO₂•)

RO₂• are an important class of ROS primarily generated within cells through lipid peroxidation chain reactions, particularly in the regions rich in unsaturated fatty acids located in cell membranes [62]. Unlike •OH, RO₂• are relatively stable due to the presence of an oxygen group, making them highly diffusible. Act as continuators and promoters (catalysts) of lipid peroxidation chain reactions, RO₂• facilitate the generating of additional lipid peroxidation products, such as H_2_O_2_, which compromises cell membrane integrity and disrupts cellular signaling [62]. They also oxidize proteins and DNA [63], leading to several structural damage, functional impairments, and genetic mutations that ultimately affect cellular and organismal homeostasis [64]. RO₂• play a significant role in oxidative stress and apoptosis, particularly in diseases, promoting cell death and tissue damage [65]. Moreover, the presence of RO₂• can enhance the generation of RO•, further exacerbating cellular damage.

##### Alkoxyl radicals (RO•)

RO• are a class of organic radicals that emerge from the decomposition of RO₂•, predominantly formed during lipid metabolism and oxidative stress responses, especially in lipid-rich areas such as cell membranes and mitochondria [66]. RO• exhibit potent oxidative activity, abstracting hydrogen atoms and reacting with biomolecules, thereby causing membrane damage and functional impairment, as well as modulating protein functionality and genomic stability [67]. These actions disrupt cellular signaling and are associated with the pathological progression of various diseases, including cardiovascular and neurodegenerative diseases. For instance, an increase in RO• level is closely related to neuronal apoptosis, which impinges on the function and health of the nervous system, underscoring the need for further mechanistic studies [68].

##### Carbonate radical anion (CO_3_•^−^)

CO_3_•^−^ is primarily generated via enzyme-catalyzed single-electron oxidation of bicarbonate or the reaction of CO₂ with peroxynitrite [69]. CO_3_•^−^ is a potent ROS and serves as an important oxidant of nucleic acids in physiological environments. Notably, CO_3_•^−^ can efficiently oxidize the guanine base of DNA through single-electron transfer reactions, yielding stable guanine oxidation products [70].

#### Non radicals

##### Hydrogen peroxide (H_2_O_2_)

H_2_O_2_ is an important ROS molecule primarily produced intracellularly through enzymatic reactions, notably by NOX family enzymes which are key dedicated generators of ROS [71]. These transmembrane enzymes utilize NADPH to reduce O_2_, directly producing H_2_O_2_ (as by NOX4, DUOX1/2) [72] or indirectly via •O_2_^–^ generation (by NOX1-3) [73], which rapidly dismutates to H_2_O_2_. Besides NOX, H_2_O_2_ is also generated in peroxisomes, mitochondria, and the endoplasmic reticulum [74, 75]. Functionally, H_2_O_2_ serves as a signaling molecule in cellular transduction, regulating processes such as cell proliferation and apoptosis, while participating in immune defense by aiding pathogen elimination [76]. However, elevated H_2_O_2_ is associated with oxidative stress, causing damage to cell membranes, proteins, and DNA, thereby linking it to pathologies including cancer and neurodegenerative diseases [47].

For instance, H_2_O_2_ mediates apoptosis through multiple pathways, including the mitochondrial pathway, protease activation pathway, and transcription factor pathway. In the mitochondrial pathway, H_2_O_2_ increases calcium ion concentrations within the mitochondria [77], regulates Bcl-2 family protein expression and activity, leading to decreased mitochondrial membrane potential, cytochrome c release, and ultimately triggering apoptosis [78]. It also oxidizes thiol groups of proteins, forming disulfide bonds that alter Bcl-2 family protein conformation and function, disrupting the balance between pro-apoptotic and anti-apoptotic proteins [79]. In the protease activation pathway, H_2_O_2_ activates cysteine proteases and other caspase family proteases, particularly caspase-3 [80]. It oxidizes thiol groups of proteases, facilitating enzymatic activation and downstream substrate cleavage, leading to apoptosis [81]. In the transcription factor pathway, H_2_O_2_ regulates the activity of transcription factors, including NF-κB, AP-1, HIF-1α, and FOXO3 [82, 83], altering gene expression and promoting apoptosis. It oxidizes thiol groups of transcription factors, affecting their DNA binding ability and transcriptional activity, leading to changes in apoptosis-related gene expression. It is worth noting that factors influencing H_2_O_2_-induced apoptosis include the concentration, the duration of action of H_2_O_2_, and the cell type. Moderate concentrations of H_2_O_2_ promote apoptosis, while high concentrations may lead to necrosis [81]. Different cell types have varying sensitivities to the duration and concentration of H_2_O_2_ treatment [84], which may be related to specific signaling pathways and antioxidant defense mechanisms within the cells [85]. Understanding these factors is of great significance for studying the mechanisms of H_2_O_2_-induced apoptosis and developing relevant therapeutic strategies.

##### Singlet oxygen (^1^O₂)

^1^O₂ is a high energy state of oxygen characterized by its potent oxidative capacity and propensity for reacting with various biomolecules [86]. This high-energy state is primarily generated through the excitation of photosynthetic pigments and photochemical reactions during photosynthesis, which occurr in chloroplasts, cytoplasm, and membrane regions within plant cells [87]. Notably, ^1^O₂ exihibits a high degree of chemical reactivity, allowing it to directly target lipids, proteins, and DNA in cell membranes, thereby inducing oxidative damage [88]. Moreover, the strong oxidizing potential of ^1^O₂ makes it an essential component in photodynamic therapy, where it is utilized to kill cancer cells or bacteria. Additionally, ^1^O₂ may influence the efficiency of photosynthesis and plant health [89].

##### Ozone (O_3_)

O_3_ is a highly reactive oxidizing gas primarily found in the upper atmosphere of the Earth. However it can enter biological systems through photochemical reactions associated with air pollution [90]. Although it does not normally exist within cells, O_3_ can significantly affect cells through respiratory exposure, particularly impacting epithelial cells and alveolar cells in the respiratory system. The strong oxidative properties of O₃ can damage cell membranes, proteins, and DNA, leading to oxidative stress and inflammatory responses that may result in respiratory symptoms as well as chronic respiratory diseases [91].

##### Lipid peroxides (LOOH)

LOOH are molecules generated from lipid peroxidation reactions, primarily localized in lipid-rich structures such as cell membranes and liposomes, particularly during periods of oxidative stress [92]. LOOH yields free radicals (LOO•) that perturb membrane structure and function, thereby initiating a chain reaction of lipid peroxidation propagated by LOO•, ultimately leads to cell membrane damage. LOOH also participates in regulating oxidative stress-related signaling pathways, influencing apoptosis and antioxidant defense mechanisms [93], underscoring its critical role in cardiovascular diseases such as atherosclerosis and in neurodegenerative diseases, triggering tissue damage and pathological progression [93].

##### Hypothiocyanous acid (HOSCN)

HOSCN is a relatively mild oxidant generated through the catalyzed reaction between H_2_O_2_ and thiocyanate (SCN^−^) mediated by peroxidases, such as myeloperoxidase. It exerts antimicrobial effects in the immune system by rapidly oxidizing bacterial proteins to combat invading microorganisms. In addition, HOSCN plays an important role in cell signaling, regulating mitochondrial function, membrane permeability and ATP production. Furthermore, it is involved in the pathogenesis of various diseases, including chronic inflammatory diseases, atherosclerosis and thrombosis. It promotes thrombosis by inducing the expression of tissue factor (TF) in endothelial cells, involving the activation of the p65/c-Rel, TF/NF-κB and ERK1/2 kinase pathways [94–97].

##### Hypochlorous acid (HOCl) and hypobromous acid (HOBr)

HOCl and HOBr are both highly reactive oxidants classified as ROS. They play essential roles in immune defense and oxidative stress processes, closely linked to the occurrence and progression of various diseases [97–99]. Neutrophils mainly produce HOCl via myeloperoxidase, while eosinophils predominantly generate HOBr through eosinophil peroxidase. These oxidants exhibit potent oxidizing properties to kill pathogens. However, an overabundance of these oxidants can lead to tissue damage thereby contributing to chronic inflammatory diseases such as asthma, chronic obstructive pulmonary disease and atherosclerosis.

### Maintenance of intracellular ROS homeostasis

ROS act as crucial signaling molecules at physiological concentrations, playing a crucial role in maintaining normal cellular functions. ROS homeostasis plays a vital role in organismal balance and physiological processes [100]. Given the abundance and diversity of ROS species present within biological systems, cells inevitably perceive and respond to distinct ROS species through specific mechanisms [101], thereby maintaining ROS homeostasis [16].

ROS function as dual agents in the cellular environment. At low–moderate levels they act as indispensable signals, whereas in excess they are cytotoxic. Brief ROS pulses support normal physiology by tuning cell-fate decisions and adaptive responses, such as aiding sperm capacitation and motility [102], priming lymphocyte activation [103], guiding stem/progenitor differentiation [104], and coordinating wound repair and angiogenesis [105]. When ROS are chronically depressed, these redox switches underperform, blunting fertility, immune activation, and metabolic plasticity. In tumors, low concentrations ROS may function as signaling molecules favoring tumorigenesis and heterogeneity [106]. Excessive ROS accumulation leads to oxidative stress. Surplus ROS attack DNA, inducing damage [107], and oxidize proteins, leading to protein misfolding and functional loss [108]. ROS also attack unsaturated fatty acids in cell membranes and lipoproteins, triggering lipid peroxidation that compromises membrane integrity and fluidity [109]. Elevated ROS themselves can act as signaling molecules, activating pro-inflammatory pathways, promoting cell death [110], amplifying inflammatory responses [111], and causing tissue damage [112]. Overall, tissue homeostasis is maintained within a bounded ROS window; both deficiency and excess are detrimental.

### The regulatory mechanism of ROS homeostasis

**Fig. 3:**
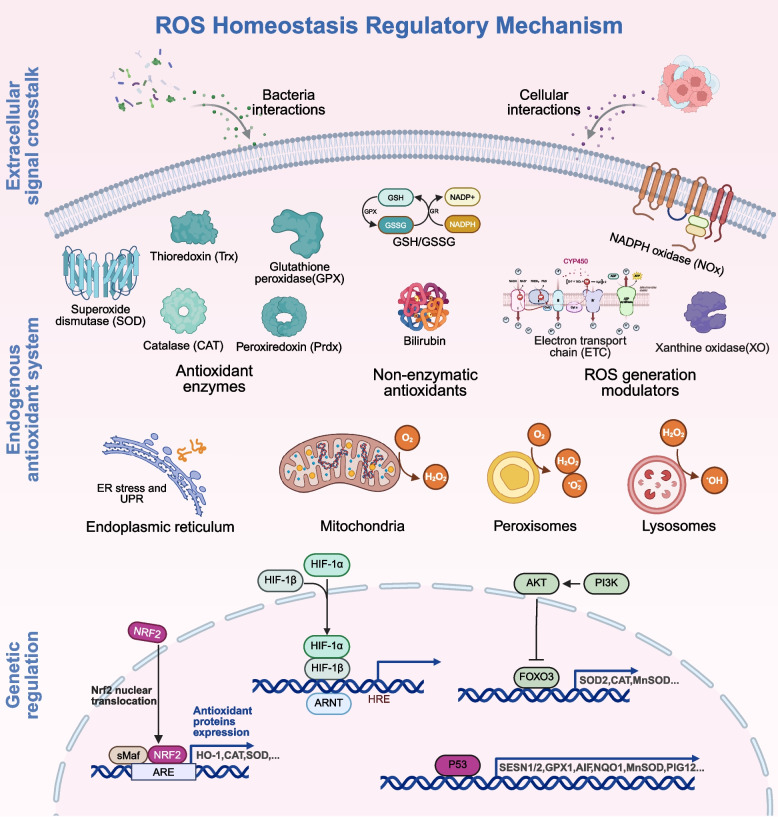
Intracellular regulatory mechanisms of ROS homeostasis. Cells possess innate endogenous antioxidant systems that regulate ROS levels through the coordinated action of endogenous antioxidants and organelles to maintain ROS homeostasis. Gene expression regulation also influences ROS homeostasis. In the extracellular environment, intercellular communication and interactions between cells and microorganisms likewise impact the ROS homeostasis of the tissue microenvironment

Faced with these challenges from ROS dysregulation, organisms employ multiple mechanisms to preserve intracellular ROS homeostasis [113] (Fig. 3). The endogenous antioxidant defense system is fundamental to this regulation. Antioxidant action primarily relies on antioxidant enzymes and non-enzymatic antioxidants [114]. SOD, located primarily in the cytosol and mitochondria, converts •O_2_^–^ into H₂O₂ and O₂ [115]. Catalase (CAT) decomposes H₂O₂ into H₂O and O₂, preventing its accumulation [116]. Glutathione peroxidase (GPx) utilizes glutathione (GSH) to reduce H₂O₂ and lipid peroxides [117]. Peroxiredoxins (Prx) employ thioredoxin (Trx) to reduce peroxides [118]. The thioredoxin system (TrxR/Trx) maintains protein thiols in a reduced state by reducing oxidized proteins (particularly disulfide bonds) and regulates the activity of redox-sensitive transcription factors like NF-κB [119]. GSH, a classic non-enzymatic antioxidant, directly scavenges ROS, and the GSH/GSSG ratio is a key indicator of cellular redox status [120]. Uric acid serves as a major plasma antioxidant, scavenging ^1^O₂ and •OH [121]. Bilirubin is a potent lipophilic antioxidant that inhibits lipid peroxidation [122].

Organisms also regulate ROS generation. The NOX family precisely controls the spatiotemporal production of ROS through multi-layered mechanisms, including subunit assembly [123], phosphorylation [124], regulation of protein stability [125], and SUMOylation [126]. This family acts as a "double-edged sword" in immune defense, cellular signaling, and oxidative stress [127]. During the sequential oxidation of hypoxanthine to xanthine and then to uric acid, xanthine oxidase (XO) incompletely reduces O₂, generating •O_2_^–^ and H₂O₂ [128]. As a core component of microsomal mixed-function oxidases, cytochrome P450 (CYP450) catalyzes substrate monooxygenation reactions. During this process, partial electron transfer via NADPH-CYP450 reductase to the CYP450-substrate complex results in "electron leakage", leading to the incomplete reduction of O₂ to •O_2_^–^ or H₂O₂ [129].

Transcriptional regulation also influences ROS levels. The NRF2/ARE pathway is a well-established antioxidant response pathway. Increased intracellular ROS oxidize cysteine residues on KEAP1, leading to NRF2 release and nuclear translocation [130]. NRF2 then binds to antioxidant response elements (ARE) [131], inducing the expression of various antioxidant genes such as SOD, CAT, and HO-1 [132, 133]. HIF-1 is a prominent pathway recently implicated in ROS homeostasis. It establishes cellular antioxidant defense by transcriptionally activating antioxidant genes (e.g., SOD2, CAT, GCL) and reductive molecules (e.g., GSH, Trx), while downregulating pro-oxidant molecule synthesis, thereby directly scavenging or neutralizing excess ROS [134]. Furthermore, HIF-1 reduces mitochondrial ROS production at its source by suppressing the expression and activity of mitochondrial respiratory chain complexes and activating glycolysis [135]. Notably, HIF-2α, another key HIF family member also regulated by hypoxia and sharing some target genes, may exhibit distinct roles from HIF-1α in ROS regulation, sometimes even promoting ROS generation under specific conditions, highlighting the nuanced complexity of this family in ROS homeostasis [136]. Other pathways, including p53 [137] and FOXO signaling pathway [138], also regulate ROS homeostasis through mechanisms involving SOD and other factors. Some clock genes are reported to directly bind the SOD1 promoter, enhancing nocturnal antioxidant defense, or regulate NADPH-generating enzymes to maintain rhythmic reducing power [139]. These circadian clock genes significantly influence the adaptive ROS threshold across different circadian phases.

Communication networks within the cellular environment also maintain ROS homeostasis. Organellar communication coordinates intracellular ROS regulation. Mitochondria, endoplasmic reticulum (ER) and lysosomes can independently sense ROS levels and regulate local ROS microenvironment stability [140]. Mitochondria possess dual mechanisms for ROS regulation [141]. On the one hand, complexes I and III of the electron transport chain are primary ROS generation sites, producing •O_2_^–^, particularly under conditions of electron leak and high membrane potential [142]. On the other hand, mitochondria can effectively reduce ETC-derived ROS by activating uncoupling proteins, which lower the inner membrane potential – a key driver of ROS production, thereby preserving cellular redox homeostasis [142]. The unfolded protein response within ER activate the PERK-NRF2 axis, inducing antioxidant gene expression to alleviate oxidative stress [143]. The ER oxidase Ero1α generates H₂O₂ as a byproduct during disulfide bond formation. Its excessive accumulation causes oxidative damage and requires precise regulation [144]. In lysosomes, the Fenton reaction involving iron ions produces •OH, but the presence of GPX4 helps prevent membrane lipid peroxidation [145]. Intercellular signal crosstalk also modulates ROS homeostasis within cellular microenvironments. Tumor-associated fibroblasts secrete cytokines such as TGF-β and IL-6, which activate NOX2 in monocytes, thereby increasing •O₂⁻ production. This ultimately leads to significantly elevated ROS levels in monocyte-derived macrophages [146]. Melatonin, a hormone secreted by the pineal gland, binds to and activates the Tyr239 phosphorylation site of the aryl hydrocarbon receptor. This enhances the expression and activity of NRF2, resulting in significantly reduced ROS levels in lipopolysaccharide (LPS)-activated microglial cells [147]. Interactions between cells and environmental microorganisms also impact ROS levels. For example, certain probiotics (e.g., *lactobacilli*) reportedly activate the NRF2 pathway via metabolic routes [148], while some pathogens (e.g., *Salmonella*, *Helicobacter pylori*) activate NOX1, upregulate ROS, and induce inflammation [149].

## ROS and oxygen homeostasis in cell fate

Firstly, it is evident that ROS plays a crucial role in maintaining oxygen homeostasis within cells according to the diverse responses and processing of ROS by cells [150, 151]. This has immediate consequences for various molecular biological processes such as intracellular signal transduction [152, 153], organelles function [154, 155], and energy metabolism [156, 157]. As such, ROS influences a variety of cellular processes including cell metabolism [158, 159], proliferation [160], differentiation [161], and death [162, 163].

### ROS homeostasis in cellular metabolism

**Fig. 4:**
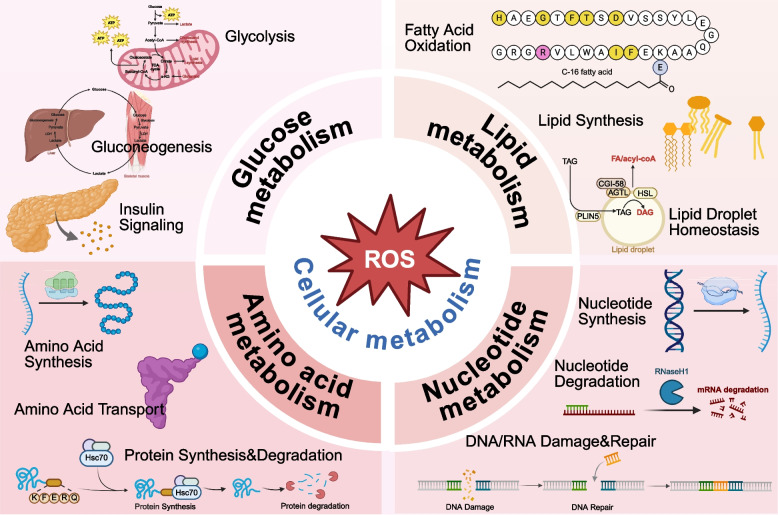
ROS affected cellular metabolism. ROS regulate key metabolic pathways in cells, including glucose metabolism, lipid metabolism, amino acid metabolism and nucleotide metabolism

The cellular response to ROS plays an important role in determining metabolic adaptations, significantly influencing physiological processes [164]. ROS regulate the activity and subcellular localization of various enzymes and transcription factors through the reversible oxidation of redox sensitive amino acid residues [164], thereby exerting a profound impact on diverse cellular metabolic processes. Mitochondria, as the primary site of ROS production, play a unique role in maintaining ROS homeostasis and energy metabolism [165]. Therefore, ROS is not only involved in ATP synthesis but also related to the balance of mitochondrial function and cellular energy supply [154]. Disruptions to this equilibrium can lead to oxidative stress, mitochondrial dysfunction, and aberrant energy metabolism [150]. Moreover, ROS is also extensively implicated in glucose metabolism [156], lipid metabolism [166], amino acid metabolism [167], nucleotide metabolism [154] (Fig. 4).

#### Glucose metabolism

The multifaceted regulatory role of ROS in glucose metabolism encompasses diverse mechanisms. By modulating key enzymes and signaling pathways, ROS significantly affects core processes such as glycolysis [168], gluconeogenesis [169], and the insulin signaling pathway [170]. Specifically, ROS can modulate the activity of key enzymes in the glycolysis process through oxidation modification, such as hexokinase [171], phosphofructokinase-1 [172], and pyruvate kinase [173]. Notably, the concentration-dependent effects of ROS on glycolysis are distinct. Low concentrations of ROS typically activate glycolysis, whereas high concentrations often inhibit it [174, 175]. In the process of gluconeogenesis, H_2_O_2_/•O₂⁻ can promote this process by activating FOXO transcription factors, which involves regulating expression levels of key enzymes such as glucose-6-phosphatase, thereby promoting glucose production [176]. Conversely, H_2_O_2_/•O₂⁻ can exert an opposite effect through the AMPK pathway, inhibiting gluconeogenesis while promoting glucose uptake and utilization [177, 178]. In regards to the insulin signaling pathway, high concentrations of H_2_O_2_/•O₂⁻ inhibit insulin receptor substrates and the PI3K/AKT pathway through oxidative stress, leading to the development of insulin resistance [179, 180]. Insulin resistance is a hallmark feature of type 2 diabetes, closely related to H_2_O_2_/•O₂⁻ levels [181]. In contrast, substances based on •O₂⁻ clearance, such as antioxidant enzymes, can play a positive role in protecting the insulin signaling pathway [182]. This is crucial for maintaining normal cellular glucose metabolism and preventing the occurrence of related metabolic diseases.

#### Lipid metabolism

ROS significantly impacts cellular lipid metabolism by modulating fatty acid synthesis, decomposition, and oxidation [166], lipid catabolism [183, 184], and cholesterol metabolism [174]. This regulation influences the overall cellular lipid metabolism process [185]. Initially, H_2_O_2_/•O₂⁻ can suppress acetyl-CoA carboxylase activity through oxidation modification, thereby reducing fatty acid synthesis [186, 187]. Additionally, it inhibits fatty acid synthase expression by activating the AMPK pathway, thus inhibiting fatty acid synthesis [188, 189]. Conversely, H_2_O_2_/•O₂⁻ promotes CPT1 expression by activating PPARα, further enhancing fatty acid oxidation [190]. Notably, low concentrations of •O₂⁻ can also regulate mitochondrial function by activating PGC-1α, thereby enhancing fatty acid oxidation [191, 192]. Lipid storage is essential for energy metabolism in living organisms. H_2_O_2_/•O₂⁻ can promote lipolysis and release free fatty acids by activating hormone-sensitive lipase [193]. Its regulation of lipid droplets has also been widely reported [194]. In cholesterol metabolism, H_2_O_2_/•O₂⁻/•OH/LOOH inhibit cholesterol synthesis by oxidatively modifying HMG-CoA reductase [195, 196]. Furthermore, during low density lipoprotein oxidation, H_2_O_2_/•O₂⁻/HOCl/•OH facilitates its conversion to oxidized low density lipoprotein, which is related to the development of atherosclerotic diseases [197].

#### Amino acid metabolism

The role of H_2_O_2_/•O₂⁻/•OH in amino acid metabolism is also particularly important. Amino acids, as the basic units of protein synthesis, are crucial for cellular function. Disruptions in their metabolic processes can be detrimental to overall cell function [198, 199]. Initially, H_2_O_2_/•O₂⁻/•OH/RO•/RO_2_• can modulate amino acid synthesis and metabolism by directly influencing the activity of amino acid synthesis enzymes, thereby regulating the utilization and synthesis of amino acids by cells [200]. Additionally, H_2_O_2_ can affect the transport of amino acids. Studies have shown that ROS can oxidize the cysteine residues in the amino acid transporter LAT1, which can affect its function and thus influence the entry of amino acids into cells [201, 202]. As a result, ROS’s impact on amino acid metabolism inevitably extends to the synthesis and degradation of proteins. For example, H_2_O_2_/•O₂⁻/•OH can oxidize amino acid residues in proteins, leading to changes in protein structure and function [203]. Notably, the impact of H_2_O_2_/•O₂⁻/•OH/^1^O₂ on the function of many proteins has been extensively reported, involving various molecular mechanisms over time [36, 101].

ROS participate in the epigenetic modification processes of proteins. Specifically, ROS regulate various epigenetic marks, including DNA methylation, histone modification, and chromatin remodeling. Moreover, H_2_O_2_/•O₂⁻/•OH modulate metabolic pathways, such as the tricarboxylic acid cycle and mitochondrial function, thereby indirectly influencing the availability of essential metabolites required for epigenetic enzyme modifications [164]. In the innate immune response, H_2_O_2_/•O₂⁻ can regulate the expression of the transcription factors involved in redox reactions, such as NF-κB, NRF2, and HIF-1, thereby mediating redox-based epigenetic modifications [204]. These studies show that ROS affects protein function and intracellular signaling through oxidative modification, thereby participating in protein metabolism and the epigenetic modification of amino acids. Meanwhile, ROS is also involved in maintaining protein homeostasis, affecting ribosome biogenesis, mRNA translation, endoplasmic reticulum oxidative folding, and protein degradation through the ubiquitin-proteasomes and autophagy pathways. For instance, H_2_O_2_/•O₂⁻ regulates protein synthesis by affecting key factors such as eIF2 and mTORC1, whereas protein folding is affected through disulfide bond formation in the endoplasmic reticulum mediated by enzymes such as PDI and ERO1A [205]. These complex regulatory networks collectively maintain the ROS balance within cells, which is crucial for cell survival, function, and metabolic regulation. In summary, ROS plays a multifaceted regulatory role in amino acid metabolism, encompassing synthesis and modification, thereby exerting a profound impact on cell function and survival.

#### Nucleotide metabolism

The significant influence of ROS on cellular nucleotide metabolism has been widely reported. First, H_2_O_2_/•O₂⁻/•OH can regulate the expression of genes related to nucleotide metabolism by transcriptionally regulating signaling pathways such as NRF2 and p53, thereby affecting the synthesis and degradation of nucleotides [206]. For example, H_2_O_2_/•O₂⁻/•OH/^1^O₂ can impair DNA synthesis substrate function by oxidizing nucleotide precursors, including dATP and dGTP [207]. Moreover, research is increasingly focused on the role of H_2_O_2_ in DNA and RNA damage repair [208, 209]. Directly attacking DNA, H_2_O_2_/•O₂⁻/•OH/^1^O₂ can cause DNA strand breakage and disrupt DNA damage repair pathways [210]. ROS can promote the oxidative damage of DNA and free deoxyribonucleoside triphosphates, leading to cell death. The MYC family of oncoproteins drives tumor development in various cancers by enhancing nucleotide synthesis and H_2_O_2_/•O₂⁻/•OH production. Through the NOX4-ROS pathway, MYC increases nucleotide oxidation, while NUDT1 depletion exacerbates this process, resulting in cell death. While most RNAs are single stranded molecules, ROS can still affect their stability and function by oxidizing RNA [211]. In conclusion, these findings demonstrate the far-reaching consequences of ROS on the fundamental genetic molecules of life, encompassing synthesis, decomposition, and damage repair. The regulatory role of ROS in cell survival and death provides promising therapeutic targets.

### ROS homeostasis in cell survival and death

**Fig. 5:**
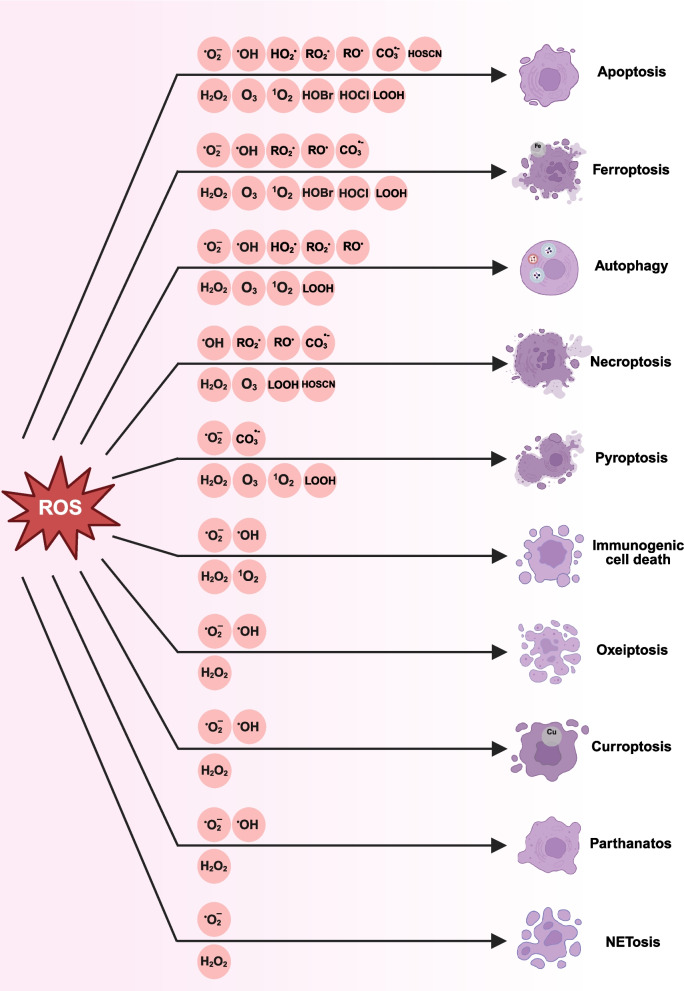
Different types of ROS are involved in different types of cell death. The ROS types above the arrows represent the free radicals involved in the death mode, while those below the arrows represent non-radical ROS

The homeostasis of ROS within cells has a profound impact on cell fate, encompassing self-renewal, proliferation, differentiation, metabolic processes, and epigenetic landscape, all of which are affected by the oxygen environment and ultimately determine the process of cell death [212]. Interestingly, sustained activation of ROS can trigger various forms of programmed cell death, including apoptosis, ferroptosis, pyroptosis, necroptosis, oxeiptosis, NETosis, parthanatos, autophagy, necrosis and immunogenic cell death [22, 213, 214] (Fig. 5).

#### Apoptosis

Apoptosis was first described in 1972 [215], representing a fundamental mechanism for maintaining cellular homeostasis. Studies have shown that H_2_O_2_/•O₂⁻/•OH regulate apoptosis and can, in turn, promote its occurrence [216]. On one hand, elevated ROS levels can precipitate oxidative stress, leading to macromolecular damage and subsequent activation of apoptotic pathways, such as the p53-mediated tumor suppress pathway [217]. Conversely, H_2_O_2_/•O₂⁻ have been shown to impair antioxidant enzymes function, thereby diminishing the cell's defenses against oxidative damage and amplifying oxidative stress [100]. Additionally, ROS activate various signaling pathways. For example, •O_2_^–^ triggers the intracellular p38 MAPK and JNK pathways, promoting apoptosis while also generating other ROS (such as H_2_O_2_) to further amplify oxidative damage [218]. In Parkinson's disease, NRF2 agonists (such as simvastatin) enhance the neuronal antioxidant capacity by upregulating antioxidant enzyme levels, thereby reducing ROS-induced apoptosis [219].

#### Ferroptosis/Cuproptosis

Since its identification in 2012, ferroptosis has been extensively linked to the involvement of •OH/LOOH [214]. The primary mechanism underlying ferroptosis is the accumulation of iron and lipid peroxidation, leading to a perturbation of oxidative homeostasis within the cellular microenvironment [220]. This process is initiated by Fe^2+^-catalyzed lipid peroxidation process through non-enzymatic (Fenton reaction) and enzymatic mechanisms (lipoxygenase) [221]. Antioxidant systems such as NRF2/HO-1/GPX4 can inhibit lipid peroxidation during ferroptosis [222, 223]. Critically, supersulfides, such as glutathione persulphide (GSSH), directly scavenge ferroptosis-implicated free radicals, notably LOO•, via rapid hydrogen-atom transfer [224], while concurrently suppressing key ferroptotic drivers including inflammatory cytokines [225] and lipid peroxidation end-products (4-HNE) [226]. Recent studies suggest that ROS play a similar role in cuproptosis as in ferroptosis [227]. When iron and copper ions are released from proteins, they can contribute to the generation of •OH, with certain chelators acting as enhancers and others functioning as inhibitors [228]. In principle, using inhibitory chelators would be a great strategy to prevent •OH production. However, due to the essential nature of iron for many biological processes, chelation therapy is typically reserved for preventing iron overload in patients requiring frequent blood transfusions, such as those with sickle cell disease and thalassemia [229].

#### Pyroptosis

Pyroptosis, a type ofprogrammed cell death, relies on inflammasome activation, characterized by cellular swelling, membrane disruption, and the secretion of pro-inflammatory cytokines [230]. Elevated ROS levels can trigger NLRP3 inflammasome activation, subsequent caspase-1 activation and gasdermin D (GSDMD) cleavage, ultimately inducing pyroptosis. The N-terminal GSDMD fragment creates transmembrane pores, leading to the initiation of pyroptotic cell death. ROS-mediated oxidative stress promotes mitochondrial dysfunction, fostering a self-reinforcing cycle where increased ROS generation perpetuates lipids, proteins and DNA oxidation, thereby exacerbating cellular injury [231]. Antioxidant systems like SOD and glutathione peroxidase (GPx) reduce •OH generation by scavenging •O_2_^–^ and H₂O₂, thereby lowering oxidative stress and inhibiting pyroptosis [232]. In non-alcoholic steatohepatitis, NRF2 agonists can reduce hepatocyte pyroptosis by enhancing antioxidant enzyme levels [233]. Collectively, these findings underscore the importance of ROS in modulating pyroptotic cell death.

#### Necroptosis

Necroptosis is a regulated form of programmed cell death that is initiated by specific death signals (such as TNF-α), which activates RIPK1 and RIPK3, ultimately leading to cell membranerupture via MLKL protein activation [234, 235]. ROS are closely related to myocardial necroptosis. The ability of H_2_O_2_/•O₂⁻/•OH to enhance the stability of the RIPK1/RIPK3 complex also promotes the occurrence of diabetes [236]. Under conditions of oxidative stress, excess H_2_O_2_ and •O_2_^–^ can increase the phosphorylation levels of RIPK1 and RIPK3, activating MLKL and inducing membrane rupture [237]. Moreover, ROS-mediated mitochondrial dysfunction exacerbates cell damage. N-acetylcysteine (NAC) and GSH, with antioxidant properties, can scavenge ROS during ischemia–reperfusion injury. The use of RIPK1 inhibitor (Necrostatin-1) [238] combined with NAC can block oxidative stress by reducing H_2_O_2_/•O₂⁻/•OH/LOOH, therefore significantly decreasing myocardial cell damage and improving cardiac function [239].

#### Oxeiptosis

Oxeiptosis is a non-inflammatory form of programmed cell death regulated by ROS, primarily triggered in response to oxidative stress caused by excessive ROS accumulation [240]. It is characterized by ROS-induced dissociation of the oxygen-sensitive kinase KEAP1, activating the NRF2 signaling pathway and caspase-8-dependent cell death [241, 242]. As the triggers of oxeiptosis begin with ROS, their key role in this process is evident. Excessive ROS can oxidatively modify KEAP1, leading to its separation from NRF2, activating the expression of antioxidant genes. However, excessive NRF2 activation can trigger caspase-8-mediated oxeiptosis. The antioxidant system reduces the level of oxidative stress, stabilizes the KEAP1/NRF2 system, and inhibits the occurrence of oxeiptosis by clearing •O_2_^–^ and H₂O₂ [243]. In chronic obstructive pulmonary disease, excessive O_3_ lead to oxeiptosis in lung epithelial cells. The use of antioxidants or compounds that upregulate NRF2 can reduce H_2_O_2_/•O₂⁻/•OH levels, thereby protecting lung cells and further improve the symptoms of chronic obstructive pulmonary disease [244].

#### NETosis

NETosis is a distinctive form of programmed cell death exclusive to neutrophils, which captures and kills pathogens by releasing a network of DNA, histones, and antimicrobial proteins (neutrophil extracellular traps, NETs) [245]. •O₂⁻ are essential for both the formation of NETs and the process of NETosis. Studies have shown that excessive HOCl/•OH generation during neutrophil activation induces extensive DNA damage and subsequent DNA repair mechanisms which will result in NETosis [246, 247]. Initially, NOX2 activation generates significant quantities of H_2_O_2_/•O₂⁻, which subsequently activate key signaling pathways such as MAPK and JNK, leading to nuclear membrane breakdown and chromatin decondensation, culminating in NET formation. Secondly, HOCl/•OH can activate enzyme complexes involved in DNA repair (such as APE1, PARP, DNA ligase) to assemble at DNA damage sites, promoting chromatin decondensation and further enhancing NETs/NETosis [247]. The antioxidant system in body can reduce the levels of •O_2_^–^ and H₂O₂, decreasing •OH production and inhibiting NETosis [245]. In systemic lupus erythematosus, excessive NETosis exacerbates autoimmune responses [248]. NOX inhibitors like Apocynin reduce H_2_O_2_/•O₂⁻ generation, suppress NET release, and alleviate inflammatory responses [249].

#### Parthanatos

Parthanatos is a form of programmed cell death characterized by the aberrant activation of PARP-1, typically triggered by excessive DNA damage [250]. H_2_O_2_/•O₂⁻/•OH induce severe DNA damage, leading to the overactivation of PARP-1 and subsequent triggering of parthanatos [251]. Under oxidative stress, excessive ROS such as •OH directly attack DNA, causing single-strand or double-strand breaks, activating PARP-1, consuming NAD⁺ and ATP, and generating poly(ADP-ribose), resulting in mitochondrial membrane potential loss and the release of apoptosis-inducing factor, ultimately resulting in cell death [252]. Conversely, antioxidant systems effectively scavenge H₂O₂, thereby reducing DNA damage, PARP-1 activation, and further parthanatos inhibition. In brain ischemia–reperfusion injury, using PARP-1 inhibitors (such as PJ-34, Olaparib) can mitigate H_2_O_2_/•O₂⁻/•OH accumulation, prevent neuronal damage, and enhance neurological recovery [253].

#### Autophagy

Autophagy serves as an essential mechanism for intracellular degradation and recycling, primarily used to clear damaged organelles and unnecessary proteins [254]. Its mechanisms is closely related to the ROS levels, for example, moderate levels of ROS can activate the autophagy process, while excessive ROS may lead to cell damages [255]. ROS initiate autophagy by activating the AMPK pathway and inhibiting the mTOR signal. Under oxidative stress, accumulation of ROS can activate pathways such as the JNK and the p53, further inducing autophagy, which helps clear damaged organelles, reduces oxidative damage, and protects cell survival [256]. Antioxidant enzymes such as SOD and GPx can scavenge •O_2_^–^ and H₂O₂, reducing •OH production and mitigating the negative impacts of oxidative stress on autophagy [257]. In Alzheimer’s disease, excessive ROS production inhibits autophagic function, resulting in the accumulation of neurotoxic proteins. Research indicates that NAC can regualate autophagy and promote the clearance of β-amyloid proteins [258], an effect that may relate to its capacity to scavenge one-electron oxidants [259], thereby positioning NAC as a potential therapeutic strategy for Alzheimer’s disease.

#### Immunogenic cell death (ICD)

ICD is a form of cell death that elicits a potent immune response, typically triggered by cancer treatments (such as chemotherapy and radiotherapy) [260]. H_2_O_2_/•O₂⁻/•OH are essential in initiating the ICD response by detecting oxidative stress and activating the ICD pathway. The build-up of ROS induces ER stress, causing the release of damage-associated molecular patterns (DAMPs) like calreticulin, ATP and high mobility group box 1 [261, 262]. These DAMPs draw in and activate immune cells, driving ICD. Moreover, NUAK1 inhibition causes tumor ICD through H_2_O_2_/•OH accumulation and ER stress, as NRF2, downstream of NUAK1, suppresses antioxidant gene expression [263]. NAC reduces oxidative levels but may impair antitumor immune responses mediated by ICD in certain contexts [264]. For instance, doxorubicin can increase H_2_O_2_/•O₂⁻/•OH generation, induce ICD, and activate antitumor responses. The combination of RIPK1 inhibitors such as Necrostatin-1 can further enhance this effect, providing novel therapeutic opportunities for cancer immunotherapy [265, 266].

### ROS homeostasis in stem cell maintenance and differentiation

Based on the ROS-sensing capacity of cells, stem cells characterized by high differentiation potential and pluripotency exhibit unique response mechanisms to ROS [267, 268]. This suggests that ROS exert profound and unexpected regulatory effects on these undifferentiated cellular populations. Multiple stem cell lineages, including germline stem cells (GSCs) [268], embryonic stem cells (ESCs) [267, 268], neural stem cells (NSCs) [268], hematopoietic stem cells (HSCs) [269], mesenchymal stem cells (MSCs) [270], epidermal stem cells (EpSCs) [271], induced pluripotent stem cells (iPSCs) [267] and organoid stem cells [267], have been reported to undergo ROS-mediated regulation, thereby influencing cell fate.

GSCs and ESCs are well-studied types of stem cells involved in reproductive development, characterized by self-renewal and multi-lineage differentiation capabilities [272]. GSCs, located in the gonads, can differentiate into sperm and eggs [273]. ESCs are stem cells extracted from early embryos (before the gastrula stage) or primitive gonads [274]. H_2_O_2_/•O₂⁻/•OH has shown significant impacts on their self-renewal and differentiation processes [275]. The level of ROS is lower in the microenvironment that maintains the self-renewal of GSCs, while it increases during the differentiation of GSCs. Both too high or too low levels of ROS can affect their regenerative potential, including proliferation, differentiation, and self-renewal [276]. In Drosophila testes, high H_2_O_2_/•O₂⁻ reduces the number of GSCs through the KEAP1/NRF2 signaling pathway and promotes the premature differentiation of GSCs via the EGFR signaling pathway. Conversely, low ROS levels result in excessive proliferation of GSC-like cells [277]. Similarly, PM2.5-induced increased ROS levels result in ESC cytotoxicity [278], while high osmotic stress mediated H_2_O_2_/•O₂⁻/•OH accumulation contribuites to ESC reprogramming [279]. The expression of antioxidant genes regulates H_2_O_2_/•O₂⁻ level in stem cell metabolism, thereby preserving their pluripotency [280]. Therefore, the balance of ROS is crucial for GSCs and ESCs to maintain their stem cell activity and differentiation capacity.

Adult stem cells, including NSCs, HSCs, MSCs, and ESCs, serve as progenitor cells for their respective tissues and organs, possessing the ability to differentiate into specialized functional cells [281]. For example, NSCs can differentiate into neurons, astrocytes, and oligodendrocytes [282], while HSCs have the potential to differentiate into various types of mature blood cells, including red blood cells, white blood cells, and platelets [283]. MSCs can differentiate into osteocytes, chondrocytes, and adipocytes [284]. H_2_O_2_/•O₂⁻/•OH has been reported to play an important role in a wide range of physiological processes of various adult stem cells [285], including regulating the proliferation and differentiation of NSCs, HSCs, MSCs, and ESCs through redox signaling pathways [286–288]. Dopamine signaling pathways and LRRK2 gene mutations can exacerbate mitochondrial dysfunction by interfering with H_2_O_2_/•O₂⁻ regulatory mechanisms, further impairing the plasticity and survival of NSCs [289, 290]. The dynamic changes of •O₂⁻ can also affect the functional state of hippocampal NSCs and are related to their transition from a resting state to an activated state [291]. Under pathological conditions, excessive H_2_O_2_/•O₂⁻/•OH causes dysfunction of NSCs, which in turn participates in neurodegenerative diseases such as Alzheimer's [292]. In HSCs, the level of ROS affects the transition of dormant HSCs to active HSCs through Myc-mediated processes [293]. The knockout of ATM can also lead to an increase in H_2_O_2_ within HSCs and induce excessive proliferation and differentiation of HSCs through the activation of the p38-MAPK pathway, ultimately leading to HSC exhaustion [294]. Exogenous addition of ROS promotes the adipogenic differentiation of bone marrow-derived hMSCs while inhibiting their osteogenic differentiation [295, 296], which may lead to the occurrence of diseases such as osteoporosis [297]. Increased ROS caused by ultraviolet radiation leads to DNA damage and cellular dysfunction [298]. Moderate levels of ROS play a pivotal role in promoting ESC proliferation and migration during wound healing [105]. Long-term inflammation and H_2_O_2_/•O₂⁻/•OH/^1^O₂ accumulation may damage the DNA of ESCs, increasing the risk of skin cancer [299]. In addition to its impact on epithelial cells, such as intestinal epithelial stem cells, H_2_O_2_ is also implicated in the pathogenesis of inflammatory bowel disease [300]. These findings underscore the multifaceted nature of H_2_O_2_/•O₂⁻/•OH, which not only regulate stem cell proliferation and differentiation but also influence genomic stability and epigenetic modifications, which is crucial for maintaining the stemness and pluripotency of stem cells [301].

iPSCs and organoid stem cells are artificially constructed engineered stem cells, derived from mature cells through specific technologies, and they possess the potential for self-replication and differentiation into various cell types [302, 303]. The balance of H_2_O_2_/•O₂⁻/•OH is crucial for engineered stem cells to maintain genomic stability and induce DNA damage responses [304]. NAC has been reported to reduce the adhesion, trans-endothelial migration, and proliferation of iPSCs, thereby decreasing the expression of molecules associated with these processes [305]. Furthermore, exogenous ROS inhibits the adhesion of iPSCs and induces apoptosis and senescence [284]. To combat oxidative stress, iPSCs employ a sophisticated antioxidant defense system, including superoxide dismutase and glutathione peroxidase, which are essential for protecting cells from ROS-induced damage [100]. The research on organoid stem cells encompasses disease modeling, drug screening and toxicity testing, infectious diseases, cancer, and so on [306–309]. Retinal organoids (ROs), derived from human embryonic stem cells (hESCs) or human induced pluripotent stem cells (hiPSCs), are self-forming organoids that mimic the structure and function of native retinas [310, 311]. ROS plays a significant role in the development and function of ROs, especially in simulating and exploring retinal diseases and drug responses. For instance, ROS may have played a key role in drug-induced retinopathy (such as the antimalarial drug chloroquine and the antibiotic gentamicin). ROS-induced oxidative stress can lead to retinal cell damage and dysfunction. The ROs, by simulating the effects of these drugs, serve as valuable platforms for studying the role of ROS in retinal diseases [312]. Studies on ROs have also found that ROS is associated with various inherited retinal diseases, such as Stargardt disease, age-related macular degeneration, or retinitis pigmentosa [313]. Therefore, maintaining a balance between ROS production and scavenging is essential for preserving engineered stem cell function and ensuring overall organismal health, underscoring their importance in regenerative medicine, disease modeling, and drug screening.

## ROS dysregulation in diseases

**Fig. 6:**
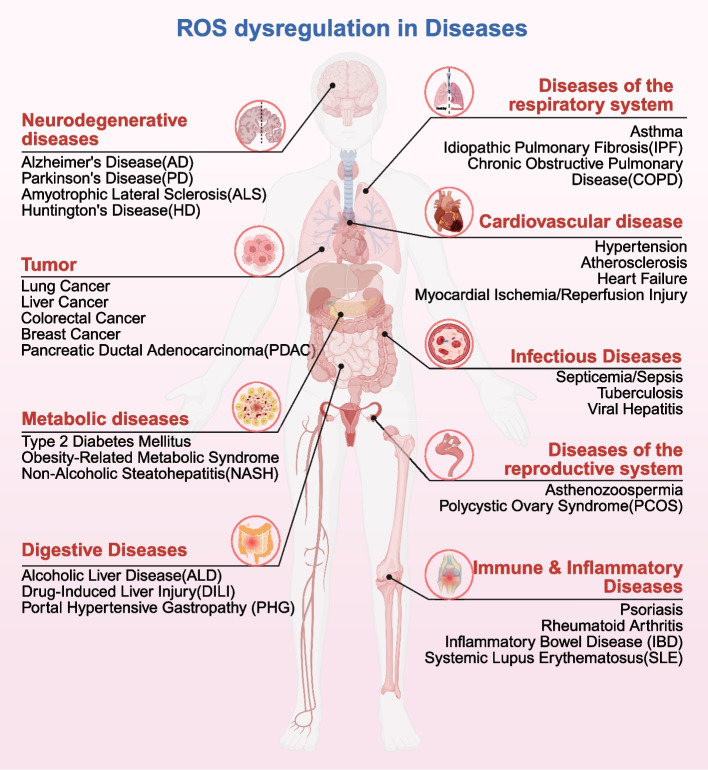
The impact of ROS homeostasis on the pathogenesis of diverse diseases, including cardiovascular diseases, neurodegenerative diseases, metabolic diseases, reproductive system diseases, digestive diseases, respiratory system diseases, cancers, inflammation and infectious diseases

ROS dysregulation has been identified as a shared pathophysiological mechanism driving the development of various diseases [100]. This dysregulation can be achieved by directly oxidizing critical biomolecules (DNA, proteins, lipids) and thereby disrupting essential cellular signaling pathways. As a result, ROS overproduction or underproduction can compromise cellular and tissue function, ultimately contributing to the pathogenesis of cardiovascular diseases, neurodegenerative diseases, metabolic diseases, reproductive system diseases, digestive diseases, respiratory system diseases, cancers, inflammation and infectious diseases (Fig. 6). Consequently, maintaining ROS homeostasis is critical for the prevention and treatment of these diseases.

### ROS dysregulation in cardiovascular diseases

ROS dysregulation represents a central node in cardiovascular diseases progression [314], involving endothelial dysfunction [315], cardiomyocyte injury [316], mitochondrial [317] and endoplasmic reticulum stress [318], vascular remodeling [319], and atherosclerosis [320]. These processes ultimately drive pathological outcomes including atherosclerosis, myocardial ischemia–reperfusion injury, heart failure, diabetic cardiomyopathy, hypertension, and arrhythmias [23].

When ROS production exceeds scavenging capacity, oxidative stress ensues—a core driver of atherosclerosis [320]. In this process, •O_2_^–^ and low-density lipoprotein (LDL) to form ox-LDL, which is phagocytosed by macrophages via scavenger receptors (LOX-1/CD36), triggering foam cell formation [321]. Concurrently, ROS impair endothelium-dependent vasodilation by inhibiting eNOS activity and accelerating NO degradation, while activating the NF-κB pathway to release pro-inflammatory cytokines [315]. This establishes a chronic inflammation-oxidative stress vicious cycle. Ultimately, activation of matrix metalloproteinases degrades collagen matrices and promotes smooth muscle cell apoptosis, leading to plaque destabilization [322]. ROS further contribute to myocardial ischemia–reperfusion injury by damaging endothelial function and promoting vascular inflammation. For instance, •O_2_^–^ reduces NO bioavailability, compromising vasodilation while activating vasoconstrictors such as endothelin-1 [315]. In cardiomyocytes, ROS activate ERK/p38 MAPK pathways to mediate hypertrophy and induce apoptosis via JNK/p53 pathways and mitochondrial membrane permeability alterations [323, 324]. Ischemia–reperfusion injury exhibits a biphasic ROS burst: xanthine oxidase activation during hypoxia synergizes with mitochondrial electron leakage during reperfusion, causing calcium overload and contractile dysfunction [325]. In heart failure, ROS impair mitochondrial electron transport chains, leading to energy metabolic failure [317]. Multiple mechanisms drive pathological ROS accumulation in diabetic cardiomyocytes, such as inhibition of mitochondrial oxidative phosphorylation elevating •O_2_^–^ production, activation of NOX by angiotensin II (Ang II) and TNF-α, and AGEs-RAGE binding-induced NF-κB activation that triggers inflammation and further ROS generation. These mechanisms collectively drive diabetic cardiomyopathy [326]. In hypertension development, Ang II activates NOX to induce O₂•⁻ bursts. ROS subsequently activate RhoA/ROCK pathways to enhance vasoconstrictor sensitivity and oxidatively inactivate protein tyrosine phosphatases [327], prolonging phosphorylation of growth factor receptors. These events synergistically promote vascular remodeling [328]. Additionally, ROS promote arrhythmias by disrupting potassium channel function, inducing endoplasmic reticulum and mitochondrial dysfunction, and causing electrical conduction disturbances between endothelial cells and cardiomyocytes [329].

### ROS dysregulation in neurodegenerative diseases

ROS dysregulation serves as a central pathological mechanism driving neuronal degeneration in multiple major neurodegenerative disorders [330]. The underlying molecular mechanisms involve direct ROS-induced oxidative damage to neuronal lipids, proteins and DNA [331], as well as ROS-mediated oxidative modifications that crucially promote the aberrant aggregation of toxic proteins [332]. This ubiquitous pathological process of oxidative stress is particularly significant in the progression of neurodegenerative disorders including Alzheimer's disease (AD), Parkinson's disease (PD), and Huntington's disease (HD).

In AD, elevated ROS levels directly promote the oligomerization and aggregation of β-amyloid peptides. This occurs partly through metal ion (Fe^2^⁺/Cu^2^⁺)-mediated redox reactions generating •OH [333]. Concurrently, ROS oxidatively modify tau protein, enhancing its hyperphosphorylation and subsequent aggregation into neurofibrillary tangles, thereby disrupting microtubule stability and axonal transport [334]. In PD, excessive ROS are deeply involved in the misfolding and aggregation of α-synuclein (α-syn) [335]. Furthermore, the dopamine metabolism process inherent to vulnerable nigrostriatal neurons intrinsically generates ROS, further damaging mitochondrial complex I and proteasome function, creating a vicious cycle of oxidative stress and neurodegeneration [336]. In HD, mutant huntingtin protein (mHTT) induces severe mitochondrial dysfunction and excessive ROS production [337]. This oxidative environment directly exacerbates mHTT aggregation and toxicity [338]. It also causes widespread oxidative damage to neuronal macromolecules, significantly contributing to the characteristic selective striatal neuronal loss in HD. Throughout these disease processes, ROS-mediated impairment of antioxidant defense systems and pro-survival pathways further amplifies the neurodegeneration cascade [339].

### ROS dysregulation in metabolic diseases

ROS play a pivotal role in the etiology of metabolic diseases such as diabetes and obesity [25]. By generating mitochondrial •O_2_^–^ bursts and promoting advanced glycation end product (AGE)-mediated molecular modifications, ROS impair pancreatic β-cell function and induce insulin resistance [340]. This subsequently contributes to the pathophysiological progression of type 2 diabetes, obesity-related metabolic syndrome, and microvascular complications (diabetic nephropathy, retinopathy).

In the pathogenesis of diabetes, sustained hyperglycemia induces mitochondrial complex III electron leakage, generating •O_2_^–^ [341]. This •O_2_^–^ surge oxidizes the PDX-1 transcription factor, suppressing insulin gene expression [342], while simultaneously activating the IRE1α-JNK pathway to trigger β-cell apoptosis [343]. Concurrently, AGEs further blunt insulin-receptor signaling via the AGEs/RAGE pathway, reducing receptor autophosphorylation and downstream transduction [340]. Furthermore, ROS-induced oxidative inactivation of PTP1B phosphatase at the Cys215 site exacerbates insulin signaling transduction impairment [344]. In diabetic nephropathy, podocyte NOX4–derived H₂O₂ drives podocyte injury and slit-diaphragm defects, thereby promoting albuminuria [345], whereas in diabetic retinopathy PKC-driven and VEGF-driven permeability changes are tightly linked to oxidative stress [346]. During obesity progression, adipose hypoxia upregulates NOX4 in white fat and couples to inflammatory cascades (including NLRP3 signaling), fostering a feed-forward ROS–inflammation loop [347]. Simultaneously, ROS oxidize the regulatory domain of PTP1B, inhibiting leptin receptor signal transduction [344], and activate the hepatic IKKβ/NF-κB pathway, inducing lipid deposition [348]. These mechanisms directly contribute to the onset and development of non-alcoholic fatty liver disease.

### ROS dysregulation in reproductive system diseases

ROS act as core effectors in reproductive dysfunction, driving pathological processes by compromising the gametogenesis microenvironment [349]. The molecular mechanisms primarily involve excessive activation of the unfolded protein response triggered by endoplasmic reticulum stress in granulosa cells [350], leading to caspase cascade-dependent apoptosis, as well as DNA strand breaks and membrane lipid peroxidation induced by mitochondrial electron transport chain leakage in spermatozoa [351, 352]. These mechanisms directly contribute to the development of reproductive disorders such as polycystic ovary syndrome (PCOS) [353] and asthenozoospermia [354].

In ovarian pathology, ROS overload in follicular fluid first activates the ER stress sensor PERK, promoting phosphorylation of the eukaryotic translation initiation factor eIF2α. This subsequently upregulates expression of the pro-apoptotic transcription factors ATF4/CHOP. This complex induces mitochondrial outer membrane permeabilization via BAK/BAX dimerization, releasing cytochrome c to trigger apoptosis [355]. Concurrently, activation of the IRE1α-JNK pathway phosphorylates the pro-apoptotic protein Bim and inhibits the anti-apoptotic function of Bcl-2, ultimately activating Caspase-3 to execute the cell death program [356]. Clinical studies confirm that the total oxidative capacity (TOC) level in the follicular fluid of PCOS patients is significantly elevated by 44.1% (P < 0.001) compared to healthy individuals, with TOC levels showing significant negative correlations with the high-quality embryo rate (r = −0.405, P < 0.01) and blastocyst formation rate (r = −0.473, P < 0.01) [357]. In male reproductive pathology, ROS generated by mitochondria in asthenozoospermic sperm cause nuclear DNA strand breaks [358] and compromise chromatin condensation integrity through aberrant histone methylation patterns, which directly correlates with diminished sperm quality [351]. Furthermore, •OH generated via the Fenton reaction oxidize polyunsaturated fatty acids in the sperm membrane, producing malondialdehyde (MDA). This lipid peroxidation impairs sperm membrane function and Ca^2^⁺ signaling, compromising CatSper-dependent hyperactivated motility [359].

### ROS dysregulation in digestive diseases

The perturbation in ROS equilibrium has been implicated as a key driver of disease pathogenesis and progression in various digestive disorders [360]. Key molecular mechanisms encompass oxidative modification of critical proteins leading to dysfunction [361], membrane lipid peroxidation disrupting organelle integrity [362], mtDNA damage causing mitochondrial dysfunction [363], and depletion of key antioxidant molecules [364]. This dysregulation significantly contributes to the development of diseases such as alcoholic liver disease (ALD), drug-induced liver injury (DILI), and portal hypertensive gastropathy (PHG).

In ALD, ethanol metabolism via hepatocyte CYP2E1 generates excess ROS and acetaldehyde, inducing endoplasmic reticulum stress and mitochondrial dysfunction [365]. ROS attack unsaturated fatty acids, initiating a lipid peroxidation chain reaction that produces reactive aldehydes. These directly damage hepatocyte membranes and mitochondria while depleting GSH, impairing detoxification capacity. This ultimately leads to steatosis, inflammatory infiltration, and hepatocyte apoptosis [366]. Regarding DILI, specific drugs (such as acetaminophen) are metabolized by CYP450 enzymes into reactive electrophiles. These directly deplete GSH, causing disruption of mitochondrial Ca^2^⁺ homeostasis and inhibition of electron transport chain complexes. This results in a surge of •O_2_^–^ and opening of the mitochondrial permeability transition pore, triggering collapse of the mitochondrial membrane potential, release of pro-apoptotic factors, and massive hepatocyte necroptosis [367]. Notably, elevated ROS levels also play a pivotal role in non-parenchymal cell damage within the digestive system, as exemplified in PHG associated with cirrhosis [368, 369]. Chronic portal hypertension drives gastric mucosal hypoxia-reoxygenation cycles and frequent iron overload in advanced liver disease. These conditions synergistically promote Fenton reactions, generating highly destructive •OH [370]. These ROS inflict direct oxidative damage on submucosal vascular cells. Protein oxidation impairs vasodilatory function and cytoskeletal integrity in endothelial cells and smooth muscle cells, while lipid peroxidation disrupts vascular membranes [371]. Furthermore, ROS act as signaling molecules inducing pathologic overexpression of VEGF and TGF-β in stromal fibroblasts [372]. This ROS-driven cascade converges on microvascular dysfunction—characterized by dysregulated vasodilation, compromised endothelial barrier function, and increased vascular fragility—ultimately manifesting as mucosal erosions and hemorrhage, the hallmark features of PHG [373].

### ROS dysregulation in respiratory system diseases

The dysregulation of ROS is a pivotal mechanism underlying the pathogenesis of various respiratory diseases, causing tissue dysfunction through direct biomolecular damage and activation of inflammatory signaling [374]. Key molecular mechanisms include ROS-mediated MAPK phosphorylation [375], triggering NLRP3 inflammasome assembly [376], oxidative modification of NF-κB inhibitor proteins leading to their nuclear translocation [377], and induction of mitochondrial DNA mutations [378]. These processes are particularly prominent in asthma, idiopathic pulmonary fibrosis (IPF), and chronic obstructive pulmonary disease (COPD).

In asthma, environmental allergen exposure triggers the activation of airway epithelial NOX. The resulting •O_2_^–^ oxidatively activates the epithelial MAPK/AP-1 pathway, promoting the release of IL-33 and TSLP. This drives Th2-type inflammation and upregulates mucin MUC5AC expression [379, 380]. Aberrant fibroblast activation in IPF depends on a ROS-TGF-β-Smad2/3 positive feedback loop. Mitochondrial ROS inactivate redox-sensitive tyrosine phosphatase PTP1B, enhancing TGF-β receptor phosphorylation [381]. This promotes α-SMA expression and collagen deposition. Concurrently, ROS-driven formation of 8-oxoG in DNA promotes epithelial-mesenchymal transition-associated transcriptional programs in epithelial cells [382]. The pathological core of COPD involves continuous ROS generation from quinones in cigarette smoke metabolized by P450 enzymes. These ROS drive NF-κB/p65 nuclear translocation, upregulating CXCL8 to recruit neutrophils. Simultaneously, they oxidatively inactivate alpha-1 antitrypsin, enhancing neutrophil elastase-mediated destruction of alveolar walls. Furthermore, mitochondrial ROS activate the NLRP3/caspase-1 axis, promoting IL-1β release and exacerbating emphysema [374].

### ROS dysregulation in cancers

ROS play a concentration-dependent dual role in cancer [383]. Low ROS levels promote oncogenic signaling transduction, while high ROS levels induce cellular damage and death [384]. Key molecular mechanisms include mitochondrial reverse electron transport activation induced by mutant proto-oncogenes KRAS/BRAF [385], oxidative inactivation of DNA repair enzymes leading to mutation accumulation [386], and constitutive activation of NRF2 pathway [387]. These processes contribute to the development, progression and metastasis of various solid tumors, including hepatocellular carcinoma, colorectal cancer, and pancreatic ductal adenocarcinoma [388].

In hepatocarcinogenesis, aflatoxin B1 (AFB1) exposure primarily induces oxidative stress through CYP3A4-mediated metabolic activation, generating ROS and MDA that directly cause DNA damage [389, 390]. Furthermore, hepatitis B virus (HBV) synergizes with AFB1 to exacerbate DNA double-strand breaks and increase TP53 R249S mutation frequency. Crucially, HBV independently suppresses DNA repair capacity by downregulating poly(ADP-ribose) polymerase 1 (PARP1) via YTHDF2-mediated m6A methylation—without enhancing ROS production or altering oxidative stress pathways [389, 391]. During colorectal cancer progression, inflammation-induced mitochondrial ROS drive malignant transformation through dual mechanisms. On the one hand, ROS induce oxidative damage to cysteine residues within the MutSα complex (MSH2-MSH6 heterodimer), specifically impairing its ATPase-dependent DNA mismatch recognition capacity. This dysfunction elevates microsatellite instability by permitting unrepaired insertion-deletion loops during DNA replication [392, 393]. On the other hand, it activates HIF-1α, which transcriptionally upregulates VEGF to promote tumor angiogenesis [394]. In pancreatic ductal adenocarcinoma, the KRASᴳ¹²ᴰ mutation induces reverse electron transport via mitochondrial complex I, generating •O_2_^–^ that activates the MAPK/ERK pro-proliferative signaling axis [395]. However, compensatory enhancement of the NRF2 pathway enables cancer cells to resist high ROS environments and develop chemoresistance [396].

### ROS dysregulation in inflammation

ROS act as both effectors and amplifiers in chronic inflammation. They are direct products of the inflammatory response and simultaneously amplify inflammatory cascades by modifying key signaling molecules [397]. Core molecular mechanisms include the specific oxidative modification of host proteins by HOCl generated via myeloperoxidase catalysis [398], the oxidative activation of NLRP3 inflammasome [399], and HIF-1α-mediated gut microbiota dysbiosis [400]. These processes drive disease progression in conditions such as psoriasis, inflammatory bowel disease (IBD), and rheumatoid arthritis.

Psoriasis, a chronic inflammatory skin disorder driven by keratinocyte metabolic reprogramming, exhibits a complex relationship with ROS that operates through interconnected pathophysiological axes [399]. Psoriasis pathogenesis involves ROS derived from upregulated NOX2/p67phox in keratinocytes, driving metabolic reprogramming via AMPK inactivation and HIF-1α stabilization. This amplifies glycolytic flux, lactate accumulation, and metabolic lactylation, fueling proinflammatory cytokine production [401]. Concurrently, ROS activate immune cells and stimulate lipid mediator generation, establishing a self-perpetuating inflammation loop through NF-κB/JAK-STAT signaling [402]. Paradoxically, while NOX2 inhibition exacerbates inflammation by disrupting redox-metabolic homeostasis, targeted activation restores metabolic balance and alleviates pathology, indicating strategic ROS modulation—not suppression—as a promising therapeutic paradigm for psoriasis. In IBD, dysregulated redox signaling and metabolic dysfunction synergistically drive disease progression. Intestinal immune cell-derived overactivation of NOX and inducible nitric oxide synthase (iNOS) generates excessive ROS, which oxidatively damage tight junction proteins in epithelial cells, compromising intestinal barrier integrity. Concurrently, this ROS burst activates the NF-κB pathway, perpetuating pro-inflammatory cytokine secretion (TNF-α, IL-1β, IL-6) and establishing a vicious cycle of chronic inflammation. This process occurs alongside impaired antioxidant defenses [397]. Critically, inflammation-induced suppression of PPARγ signaling further disrupts mitochondrial β-oxidation in epithelial cells, reducing oxygen consumption. This microenvironmental shift causes profound dysbiosis [403]. The expanded *Enterobacteriaceae* release LPS, activating the TLR4/MyD88 axis to upregulate epithelial NOX1 expression, thereby driving secondary ROS bursts that amplify epithelial barrier disruption and inflammatory cascades [404]. Within the rheumatoid arthritis joint cavity, •O_2_^–^ produced by synovial fibroblasts oxidizes cysteine 645 of protein-arginine deiminase (PAD). This oxidation enhances PAD enzymatic activity, promoting the citrullination of joint proteins. The resulting citrullinated proteins induce the production of anti-cyclic citrullinated peptide autoantibodies, amplifying autoimmune damage [405, 406].

### ROS dysregulation in infectious diseases

ROS exhibit a dual role in infectious diseases. While low-level ROS assist host defense by clearing pathogens, sustained overproduction causes oxidative stress that exacerbates tissue damage [407]. Core molecular mechanisms encompass the targeted modification of inflammatory signaling molecules [408], oxidative alteration of pathogen entry-related proteins [204], and direct damage to host lipids/DNA [409]. Significant ROS imbalance occurs in representative diseases including septicemia, sepsis, tuberculosis, and viral hepatitis.

In the pathology of septicemia, pathogen-associated molecular patterns activate NOX2 [410], triggering an electrogenic respiratory burst that pumps •O_2_^–^ into phagosomes. While •O_2_^–^ itself is not directly microbicidal, it drives critical ionic fluxes that alkalinize and hypertonify the phagosomal lumen. This environment activates neutral proteases, enabling degradation of ingested pathogens—the primary killing mechanism [411]. Concurrently, under oxidative stress, catecholamines undergo auto-oxidation to form benzoquinone derivatives. These directly inhibit mitochondrial complex III function, reducing ATP synthesis and promoting the release of apoptotic factors. In sepsis, pathogens activate neutrophils to release NETs. This process drives a burst of ROS production via NOX2, which hyperactivates the TLR4/NF-κB pathway. The resulting systemic cytokine storm coincides with ROS attacking tight junction proteins in capillary endothelial cells, inducing microcirculatory leakage [412]. During tuberculosis infection, *Mycobacterium tuberculosis* (MTB) escaping into the macrophage cytosol triggers mitochondrial ROS (mtROS) bursts. These mtROS suppress autophagic clearance by oxidatively inactivating the IRS-1/PI3K pathway [413], while simultaneously activating HIF-1α to promote glycolysis, creating a nutrient-rich microenvironment for MTB. Concurrently, excessive ROS oxidatively damage macrophage GPX4, triggering ferroptosis that disseminates infection. In viral hepatitis, HBV/HCV trigger ER stress and upregulate NOX1/NOX4, increasing ROS; KEAP1 cysteine oxidation then stabilizes NRF2 to allow its nuclear translocation, but chronic infection dysregulates this pathway, permitting ROS accumulation. Elevated ROS reinforce TGF-β/Smad signaling, driving hepatic stellate cell transdifferentiation into myofibroblasts and fibrogenesis [414].

## Therapeutic interventions targeting ROS homeostasis

As fundamental signaling molecules within cells, ROS extensively participate in the regulation of various cell fates within an organism through pathways such as cellular metabolism and signal transduction. Consequently, ROS have far-reaching impacts on the entire lifecycle of an individual, from embryonic development to growth, aging, and disease progression. Based on the crucial role that ROS play in the pathology and pharmacological treatment of a wide range of diseases, therapeutic strategies targeting the regulation of ROS homeostasis hold significant clinical value for revealing the molecular mechanisms of diseases, promoting drug development, and advancing personalized treatment. Current ROS-targeted interventions include antioxidant therapies, conventional pro-oxidant therapies, and other emerging novel approaches modulating ROS homeostasis.

### Conventional antioxidant strategies

**Fig. 7:**
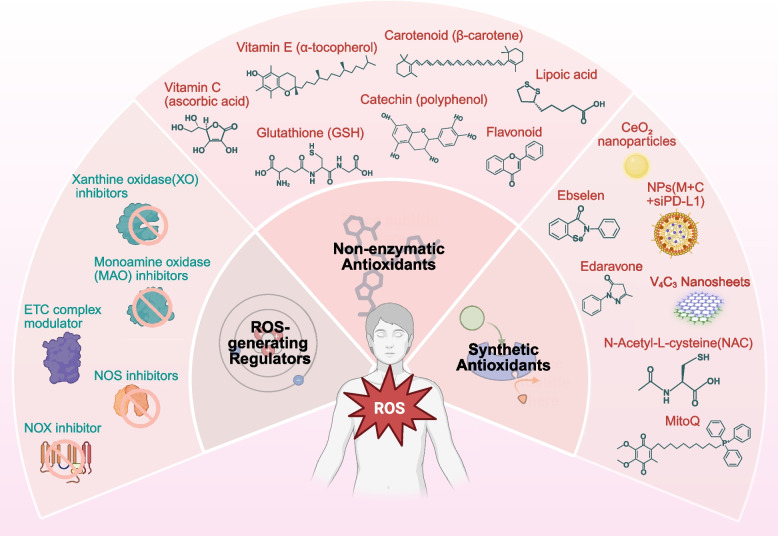
Regulators of ROS homeostasis. In addition to the body's endogenous antioxidant systems, exogenous antioxidants also cooperate to maintain ROS homeostasis. These exogenous antioxidants mainly include non-enzymatic antioxidants, synthetic antioxidants, and ROS-generating regulators

ROS-based antioxidant strategies are common therapeutic approaches that have been widely applied in both clinical and basic research fields. Current antioxidant strategies mainly include antioxidants for ROS scavenging and modulators of ROS generation (Fig. 7).

#### Exogenous antioxidants

Exogenous antioxidants constitute an important component of the body’s antioxidant defense network, acting in concert with other defense mechanisms to maintain cellular and tissue homeostasis. Exogenous antioxidants primarily originate from food and plants, including vitamins, trace elements, carotenoids, and polyphenols, among others. Vitamins, such as Vitamin C (ascorbic acid) and Vitamin E (tocopherol), possess strong antioxidant capabilities and can neutralize free radicals, protecting cells from oxidative damage. Reports have indicated that Vitamin C and Vitamin E can directly neutralize ROS, further enhancing the cell's antioxidant defense capabilities [415, 416]. The therapeutic potential of antioxidants extends beyond cellular protection, with clinical applications that enhance patient outcomes and quality of life. Trace elements such as selenium and zinc play essential roles in the body’s antioxidant defense system, as they serve as integral components of various antioxidant enzymes. Selenium is a vital cofactor for glutathione peroxidase (GSH-Px), which catalyzes the reduction of H₂O₂ to H₂O using reduced GSH, thereby mitigating oxidative damage at the cellular level. Similarly, zinc is a structural and functional component of multiple antioxidant enzymes, including SOD, which catalyzes the dismutation of •O_2_^–^ into H₂O₂ and O_2_, reducing the toxicity of free radicals. [417]. Carotenoids, such as β-carotene, lutein, astaxanthin, and lycopene, not only have antioxidant effects but can also be converted into Vitamin A, participating in a variety of physiological processes [418]. For instance, β-carotene is converted into Vitamin A through a cleavage process that is widely present in mammals. Astaxanthin, with its ability to traverse cell membranes, neutralizes free radicals and mitigates oxidative damage, thus providing protective effects against retinal degeneration [419]. Polyphenolic compounds (such as resveratrol and flavonoids) are widely found in fruits, vegetables, and tea, and have various biological activities, including antioxidant, anti-inflammatory, and anticancer effects [420–422]. Flavonoids scavenge ROS through the high sensitivity of their phenolic parts to oxidation. Although the antioxidant capacity of flavonoids may be compromised after reacting with ROS, recent studies have shown that the oxidized derivatives of certain flavonoids can significantly enhance their original antioxidant properties by activating the NRF2/KEAP1 pathway and upregulating the cell's endogenous antioxidant capacity [423].

#### Synthetic antioxidants

Synthetic antioxidants, a broad category of artificially engineered molecules, include nano-antioxidants and other chemical derivatives. While synthetic antioxidants play a pivotal role in food preservation and industrial application, their medical use remains restricted due to potential health concerns, necessitating further research into safer and more effective formulations. Therefore, the research and development of safer and more efficient synthetic antioxidants remains an important direction for research. Nano-antioxidants represent a novel class of synthetic antioxidants, which can be broadly categorized into metal oxides, metallic nanoparticles, and antioxidant-functionalized nanoparticles. Owing to their superior antioxidant capacity and excellent biocompatibility, these nanomaterials have gained significant attention in the fields of advanced materials and biomedicine, where they are employed to enhance both the stability and biocompatibility of therapeutic agents and delivery systems. Oxides such as nano-titanium dioxide (TiO_2_), nano-zinc oxide (ZnO), nano-silicon dioxide (SiO_2_), and nano-cerium oxide (CeO_2_) have a wide range of applications in the medical field. TiO_2_, known for its photocatalytic activity, is widely used in photothermal therapy and photodynamic therapy for cancer, generating ROS to kill cancer cells through light exposure [424]. ZnO excels in antibacterial and anticancer aspects. ZnO nanoparticles have good antibacterial properties, producing ROS to destroy bacterial cell walls and effectively combating a variety of bacteria [425]. In terms of anticancer effects, ZnO nanoparticles are sensitive to blood tumor cells, producing ROS to induce apoptosis, and have less impact on normal cells. CeO_2_ has garnered increasing attention for its exceptional antioxidant properties, effectively protecting normal cells from radiation-associated damage, oxidative stress, and inflammatory responses. CeO_2_ has enzyme-like properties, such as CAT and SOD, making it important in the field of tissue engineering [426]. Antioxidant-functional nanoparticles are nanoscale materials capable of modulating redox reactions to counteract oxidative stress. These nanoparticles are often biosynthesized from a variety of natural sources, including algae, bacteria, fungi, lichens, and plants. A notable example of antioxidant functional nano-platform is NP (M + C + siPD-L1), an engineered multifunctional nanoparticle prepared by encapsulating Toll-like receptor agonists, catalase, and siPD-L1. It disrupts the balance of the tumor immune microenvironment by synergistically regulating immune cells, precisely antioxidant, and relieving immune suppression, forming a microenvironment conducive to anti-tumor immunity, thereby exerting a powerful cancer immunotherapy effect and showing good biocompatibility and tumor accumulation characteristics [427, 428]. Vanadium carbide nanosheets (V₄C₃ NSs) exhibit broad-spectrum antioxidant activity, making them a promising therapeutic candidate for idiopathic pulmonary fibrosis. These nanosheets effectively scavenge ROS, thereby inhibiting the proliferation of myofibroblasts and the accumulation of aberrant extracellular matrix—key pathological features in pulmonary fibrosis. Moreover, valence engineering enables multiple antioxidant mechanisms, such as electron transfer, hydrogen atom transfer, and enzyme-mimetic catalysis, endowing V_4_C_3_ NSs with highly efficient and durable antioxidant capabilities [429]. Although research on nano-antioxidant functional nanoparticles in the biomedical field has made notable progress, their long-term biological effects and potential risks remain insufficiently understood. Further experimental and clinical investigations are essential to comprehensively evaluate their safety and efficacy, ensuring their reliable translation into clinical applications. Other synthetic antioxidants such as Ebselen, Edaravone, and NAC not only have antioxidant effects but also exert protective effects through multiple mechanisms. First, Ebselen is an enzyme mimetic initially developed to simulate the activity of GPx. It not only has antioxidant effects but also increases NRF2 levels, clears peroxynitrite, inhibits Fe^2^⁺ transport, and binds to protein thiol groups [430]. Ebselen has shown certain therapeutic effects in clinical trials for stroke patients [431]. Second, Edaravone is used to treat stroke and amyotrophic lateral sclerosis, with moderate clinical effects [432–435]. It was initially developed as a scavenger of •OH and other ROS, but may exert effects through other mechanisms in vivo [436]. Finally, NAC is mainly used to treat acetaminophen poisoning and as a mucolytic agent [437]. Although it is generally regarded as an antioxidant that maintains GSH levels by serving as a cysteine prodrug for de novo GSH synthesis [438], accumulating evidence indicates that NAC also gives rise to low–molecular-weight persulfides via H₂S/sulfane-sulfur pathways. These highly nucleophilic reactive sulfur species scavenge oxidants/electrophiles and modulate redox signaling, thereby influencing ROS homeostasis [439]. NAC has been reported to exacerbate tumor progression in certain animal models, potentially by interfering with cellular signaling pathways or reacting with lipid peroxidation products. Its mechanism of action is complex and cannot be simply characterized as a ROS scavenger. Therefore, further research is needed to elucidate its specific effects across different biological systems and pathological contexts.

#### Strategies aimed at ROS generation

Inhibitors of ROS production, including NOX inhibitors, mitochondrial ROS inhibitors, monoamine oxidase inhibitors, XO inhibitors, and NOS inhibitors, all have higher specificity [440]. For example, NOX inhibitors effectively suppress ROS production, thereby reducing oxidative stress and protecting cells from oxidative damage. Apocynin is a natural NOX inhibitor, which not only effectively reduces the generation of ROS but also enhances the effect of other antioxidants, improving the prognosis of related diseases [441]. Although not all, many classical NOX inhibitors are natural products. Consequently, they may exhibit undefined or broad specificity and relatively high equilibrium dissociation constant (Kd) values for their targets [442]. Curcumin can not only reduce the production of uric acid by inhibiting the activity of XO, alleviating the symptoms of hyperuricemia and gout to a certain extent [443, 444], but also inhibit the activity of inducible nitric oxide synthase, reducing the production of NO [445, 446]. Moreover, natural antioxidants such as carotenoids and polyphenolic compounds can also inhibit the generation of ROS. For example, lutein and lycopene protect the retina from oxidative damage by reducing the production of ROS inside and outside the cells [447]. Lycopene significantly inhibits the proliferation of prostate cancer cells and promotes their apoptosis in vitro, and can also reduce the expression of inflammatory factors in prostate cancer cells [448]. Resveratrol reduces the production of ROS through multiple mechanisms. Notably, studies on resveratrol analogs have shown that the hydrogen atom transfer mechanism is the most important mechanism for its free radical scavenging activity [449, 450]. In addition, a variety of synthetic compounds with in vitro antioxidant activity have been developed to treat diseases, including recombinant SOD, catalase, nitroxyl radicals, iron ion chelators, deuterated PUFAs, thiols, and ROS-generating enzyme inhibitors. Owing to their distinct biochemical mechanisms that suppress lipid peroxidation and reduce ROS production, these compounds have been investigated in clinical studies to assess the therapeutic value of their antioxidant properties (Table 1).

**Table 1:** Representative clinical trials of ROS generation modulators

Category	Intervention	ClinicalTrials.gov number	Diseases	Phase	Status
NOX inhibitors	Apocynin	NCT00992667	Bronchial asthma	Phase 1	Completed
		NCT04657926	Knee osteoarthritis	Phase 2	Completed
		NCT01402297	Chronic obstructive pulmonary disease	Phase 1	Completed
NOS inhibitors	L-NAME	NCT00835224	Orthostatic hypotension; Spinal cord injury	Phase 2	Completed
	L-NMMA	NCT02834403	Metastatic triple-negative breast cancer	Phase 1&Phase 2	Completed
MAO inhibitors	Safinamide	NCT05312632	Parkinson’s disease	Phase 4	Completed
		NCT03944785	Parkinson’s disease	Observational	Completed
		NCT03753763	Multiple system atrophy	Phase 2	Completed
XO inhibitors	Curcumin	NCT01964846	Inflammation	Not Applicable	Completed
		NCT01333917	Colorectal cancer	Phase 1	Completed
Polyphenols/flavonoids	Resveratrol	NCT02905799	Knee osteoarthritis	Phase 3	Completed
	Flavonoids	NCT00006504	Cardiovascular disease	Observational	Completed
	Quercetin	NCT00402623	Sarcoidosis	Not Applicable	Completed
Thiols	α-Lipoic acid	NCT06406127	Breast cancer	Phase 4	Recruiting
Enzyme mimetics	Ebselen	NCT03013400	Bipolar disorder	Phase 2	Completed
	GC4419 (Avasopasem manganese)	NCT04555096	COVID-19	Phase 2	Terminated

*NOX *NADPH oxidase, *NOS* nitric oxide synthase, *MAO* monoamine oxidase, *XO* xanthine oxidase, *ETC* electron transport chain

The clinical trials were obtained from ClinicalTrials.gov (https://www.clinicaltrials.gov/)

The NRF2/ARE pathway is a key cellular signaling pathway that plays a crucial role in regulating the cellular response to oxidative stress [451]. The classical NRF2/ARE pathway regulates redox balance via KEAP1-mediated control. Under oxidative stress, KEAP1 modifications release NRF2, enabling its nuclear translocation to activate antioxidant genes. This orchestrates ROS detoxification and cellular protection [452, 453]. Therefore, research has identified NRF2/ARE pathway activators as potential therapeutic targets for modulating ROS in disease treatment. Currently, NRF2/ARE pathway activators are mainly divided into natural and synthetic compounds [454]. Numerous natural polyphenols have been identified as NRF2 activators. For example, curcumin, an extract from the rhizome of the plant Curcuma longa mentioned above, inhibits ROS production and degrades KEAP1 to activate the NRF2/ARE pathway [455]. Resveratrol from grapes and quercetin from the Sophora japonica tree also have the ability to activate NRF2 [456]. In addition, α-spinasterol isolated from Achyranthes aspera has been shown to modulate NRF2, HO-1, and NQO1 proteins, thereby exerting antioxidant and anti-inflammatory effects [457]. Synthetic compounds such as dimethyl fumarate (DMF) and its major metabolite monomethyl fumarate are synthetic compounds that have been proven to activate the NRF2 pathway [458]. DMF has been approved as a first-line treatment for relapsing multiple sclerosis and is used in clinical treatment, with its therapeutic effects partly attributed to the activation of NRF2 [459]. The synthetic triterpenoid compound bardoxolone methyl (bardoxolone) has also been studied for potential therapeutic applications in various diseases, such as in clinical trials for the treatment of chronic kidney disease, type 2 diabetes, and Alport syndrome [460–463]. Another triterpenoid compound, omaveloxolone, has been approved by the FDA for the treatment of Friedreich's ataxia [464].

In summary, significant progress has been made in the research on NRF2/ARE pathway activators as modulators of ROS generation. A variety of natural and synthetic compounds have been identified as activators, and some of their mechanisms of action have been partially elucidated. Moreover, these findings hold significant implications for developing novel therapeutic strategies targeting the NRF2/ARE pathway as well as other antioxidative pathways such as p53 and HIF. For instance, future research may focus on exploring structure–activity relationships of NRF2 activators to identify more effective and selective compounds, while investigating their potential therapeutic applications across diverse disease models. These findings are of great importance for the development of new therapeutic strategies for various diseases related to oxidative stress. Future research may focus on further exploring the structure–activity relationship of NRF2 activators, identifying more effective and selective compounds, and studying the potential therapeutic applications of these activators in different disease models.

### Conventional pro-oxidant strategies

In addition to antioxidant strategies, several pro-oxidant regulatory approaches have long been applied in both clinical and basic research. These include platinum-based pro-oxidant drugs and HIF-mediated hypoxia regulation agents for ROS modulation, among others.

#### Promotion of oxidative stress

The approach of treating diseases by promoting oxidative stress has gradually gained attention in medical research, especially in cancer treatment. It is well established that moderate oxidative stress can trigger apoptosis in cancer cells, which has traditionally led to the use of antioxidants as a therapeutic approach. However, studies have found that antioxidants can also promote the metastasis of lung cancer [465], which also implies that oxidative stress and oxidative damage cannot be equated directly. In cancer treatment, some widely used chemotherapeutic drugs (such as cisplatin, doxorubicin, arsenic trioxide (As_2_O_3_), paclitaxel) enhance the apoptosis of cancer cells by inducing the production of intracellular ROS, prompting tumor cells to lose their ability to survive in an oxidative stress environment [466, 467]. For example, cisplatin promotes cell cycle arrest and apoptosis by causing DNA damage and oxidative stress [468, 469]. Doxorubicin, on the other hand, generates free radicals, causing intracellular oxidative damage and further activating apoptotic signaling pathways [470, 471]. In addition, As_2_O_3_ also plays an important role in the treatment of acute promyelocytic leukemia, promoting cell apoptosis by inducing ROS accumulation [472, 473]. Although these drugs have significant effects in antitumor and other disease treatments, due to their lack of selectivity, they may also cause damage to normal cells, leading to side effects and drug resistance [474]. In cardiovascular diseases, oxidative stress is closely related to the occurrence and development of atherosclerosis [475]. By moderately promoting oxidative stress, the antioxidant defense mechanisms of vascular endothelial cells can be activated, reducing the formation of oxidized LDL and thus alleviating the progression of atherosclerosis [197]. In inflammatory bowel disease, oxidative stress is closely related to intestinal inflammation. By moderately promoting oxidative stress, the antioxidant defense mechanisms of intestinal epithelial cells can be activated, reducing the release of inflammatory factors and thus alleviating intestinal inflammation [476].

In summary, moderately promoting oxidative stress has potential application value in the treatment of certain diseases. However, it should be noted that the regulation of oxidative stress needs to be very precise. Excessive oxidative stress can lead to cell damage and disease exacerbation. Therefore, caution is needed in clinical applications.

#### Managing hypoxia by HIFs

Hypoxia represents another key factor inducing cellular perception and oxidative stress [477]. Hypoxia not only manifests as the direct effect of insufficient oxygen supply to tissues, but the microenvironmental disturbances it triggers are often accompanied by significant oxidative stress, constituting a core factor exacerbating pathological damage [5]. Under low-oxygen conditions, mitochondrial electron transport chain dysfunction and hypoxia-induced activation of oxidases [478] synergistically drive pathological ROS accumulation [479] that overwhelms cellular antioxidant defenses. Crucially, this ROS surge actively drives pathology as a core hypoxic stress mediator, directly regulating inflammatory responses, cell death pathways, and autophagy via dysregulation of key signaling effectors. Thus, hypoxia-generated ROS represents a fundamental pathogenic mechanism contributing to chemoresistance [480], ischemia–reperfusion injury [481], chronic inflammation [482], and neurodegeneration [483].

**Fig. 8:**
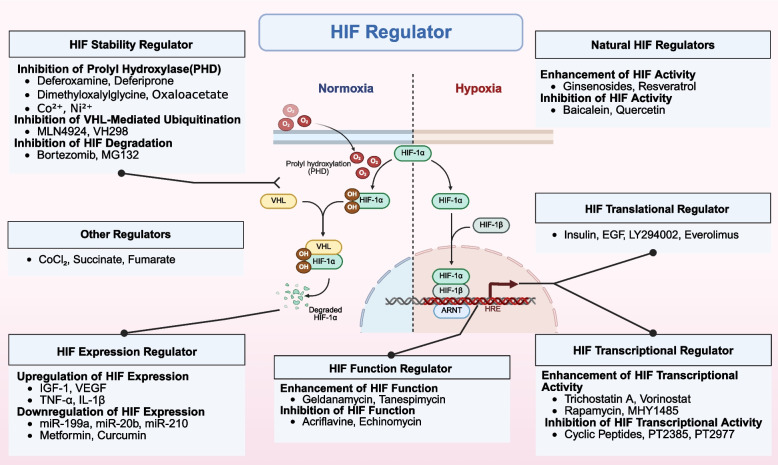
Hypoxia-targeted therapeutic strategies via HIF modulation. HIF-targeted approaches encompass HIF stability regulator, natural HIF regulators, HIF expression regulators, HIF transcriptional regulators, HIF translational regulators, HIF functional regulators

The core regulation of hypoxia adaptation is the HIF pathway, making it both a challenge and a potential therapeutic target for hypoxia- and oxidative stress-related diseases (Fig. 8). The core mechanism by which cells sense and respond to hypoxia relies on HIFs. HIFs are key transcriptional regulators mediating cellular adaptation to hypoxic environments [484], primarily regulating the expression of a series of oxygen-dependent genes [485]. Therefore, targeting the HIF signaling pathway and its regulated downstream gene expression has become a potential strategy for treating hypoxia-related diseases.

To effectively intervene in oxidative stress damage within hypoxia-related pathological processes, the core strategy targeting the HIF pathway focuses on regulating the generation and homeostasis of pathological ROS, rather than solely addressing hypoxia adaptation itself. Interventions in this direction mainly manifest in two complementary logics: one is to indirectly block hypoxia-induced pro-oxidant signaling axes by inhibiting HIF function [486]. The other is to actively utilize the hypoxic microenvironment to design prodrugs that selectively intensify local oxidative pressure [487]. On one hand, specifically blocking HIF signaling can significantly attenuate hypoxia-driven pathological ROS accumulation. Representative drugs like the highly selective HIF-2α antagonist Belzutifan (MK-6482) [488] inhibit the expression of pro-angiogenic and pro-survival genes, alleviating abnormal metabolic burden and vascular disruption in tumor tissues. This, in turn, reduces mitochondrial dysfunction and NOX activity persistently activated by chronic hypoxia, suppressing abnormal ROS bursts at the source. Conversely, while broad-spectrum PHD inhibitors (e.g., Roxadustat [489]) promote erythropoiesis to treat anemia, their potential pro-oxidant side effects also require careful evaluation, reflecting the dual nature of intervention strategies. On the other hand, the most targeted strategy involves hypoxia-activated prodrugs (HAPs) [490, 491] designed for the hypoxia-ROS microenvironment. Their core mechanism lies in being bio-reductively activated in specific low-oxygen regions, actively releasing or generating large amounts of ROS for precise killing. For example, the quinone compound Tirapazamine [492] undergoes redox cycling under hypoxia, continuously producing •O_2_^–^ and •OH, directly triggering oxidative bursts. The nitroimidazole prodrug Evofosfamide (TH-302) [493–496] releases alkylating agents upon reductive cleavage while concomitantly producing cytotoxic free radicals, synergistically exacerbating local oxidative damage. The bioreductive agent AQ4N [497, 498] significantly depletes reducing power during activation and may generate free radicals, enhancing the elimination efficiency of hypoxic cells. These strategies achieve active manipulation of ROS generation pathways by directionally amplifying oxidative pressure in lesion areas. In the research of tumor treatment, utilizing hypoxia-targeted nanocarriers to deliver drugs directly to hypoxic areas remains a promising strategy [499]. Liu et al. developed a novel hypoxia-activated combination nanomedicine that targets hypoxic tumors through hypoxia-sensitive block copolymers and photothermal/photodynamic therapy (PTT/PDT), efficiently delivering drugs and achieving synergistic anticancer effects of chemotherapy and PDT/PTT, completely eliminating advanced breast cancer, including hypoxic and metastatic tumors, stimulating a systemic antitumor immune response, and significantly improving survival rates, showing important clinical application potential [500].

In summary, the core breakthrough in current HIF-targeted drug development lies in establishing the regulation of ROS generation (inhibiting pathological accumulation or controllably utilizing its killing effect) as the key hub for intervening in hypoxia-related diseases. It should be noted that while the HIF pathway can induce antioxidant genes like SOD2 to maintain adaptive balance under acute hypoxia, this protective effect in chronic diseases like cancer may conversely aid malignant cells in resisting apoptosis, constituting another dimension of intervention complexity [501]. Future research needs to continuously deepen the mechanistic analysis of the HIF-ROS interaction network to optimize therapeutic strategies based on redox homeostasis.

### Novel approaches for modulating ROS homeostasis

As the role of ROS homeostasis in disease therapeutic interventions gains increasing attention, emerging ROS homeostasis modulation strategies are being actively explored to influence disease progression and treatment efficacy. Simultaneously, novel therapeutic targets and innovative approaches continue to be developed for clinical translation.

#### Targeting key signaling pathways in ROS homeostasis

ROS homeostasis and the related signaling pathways occupy a central position in the pathogenesis of many diseases. In-depth exploration of these pathways and their interactions is of great significance for identifying new clinical treatment targets. Beyond the extensively studied NRF2 pathway, which has been developed as a therapeutic target [502, 503], the PI3K/AKT/mTOR signaling pathway also plays a crucial role in cell survival and metabolism. Oxidative stress can activate this pathway, promoting cellular antioxidant defense and energy metabolism [504]. In diseases such as cancer and diabetes, abnormal activation of this pathway is closely related to disease progression, and modulating its activity may improve disease prognosis [505–507]. Hypoxia signaling also interacts with the PI3K-mTOR pathway and plays an important role [508]. In addition, hypoxia signaling has a significant impact on the Notch1 and HIF-1α signaling pathways, which influence each other and participate in physiological and pathological processes related to hypoxia, such as angiogenesis and tumor metastasis [509, 510]. The NF-κB signaling pathway is activated under hypoxic conditions and participates in inflammatory responses and immune regulation, becoming a potential therapeutic target for hypoxia-related diseases [511]. Vascular endothelial growth factor (VEGF) and its receptors, as key factors promoting angiogenesis, are upregulated under hypoxic conditions. Targeting VEGF and its receptors can inhibit abnormal angiogenesis, providing new ideas for the treatment of hypoxia-related diseases [512]. In conclusion, targeting key regulatory molecules in ROS homeostasis-related signaling pathways holds great promise for innovative clinical therapies.

Certainly, emerging therapeutic intervention strategies for regulating ROS—including gene therapy, stem cell therapy, and combination therapies such as antioxidant chemotherapy—have already demonstrated promising applicational potential. These therapies modulate ROS levels through different mechanisms to achieve therapeutic effects. First, gene therapy can regulate ROS levels by targeting ROS-related genes. For example, CRISPR-Cas9 gene editing technology can be used to edit genes related to ROS generation and clearance, such as NQO1, p53, and NRF2, thereby regulating ROS levels [513–515]. In addition, gene therapy can enhance cellular antioxidant capacity by upregulating the expression of antioxidant enzymes, thus reducing ROS accumulation. Second, stem cell therapy modulates ROS levels by utilizing the differentiation and repair capabilities of stem cells. For instance, MSCs can secrete various cytokines that promote tissue repair and regeneration, and also have antioxidant effects that can reduce ROS production. Furthermore, stem cells can replace damaged cells by differentiating into functional cells, thereby restoring tissue function [22]. Antioxidant chemotherapy, an emerging combination approach, involves pairing antioxidants with chemotherapeutic agents to regulate ROS levels more effectively. NAC can alleviate oxidative stress, while chemotherapeutic agents such as cisplatin induce cancer cell death by increasing ROS production. When used in combination, NAC and cisplatin may enhance the cytotoxic effects on cancer cells while simultaneously protecting normal cells by mitigating oxidative damage [516, 517], and the combination can also reduce side effects such as ototoxicity caused by cisplatin administration [518]. These emerging therapeutic intervention strategies provide new strategies for regulating ROS levels and hold promise for playing important roles in the treatment of cancer and other diseases. Future research should further explore the mechanisms and applications of these therapies to develop more effective treatment plans.

#### Adaptive regulation strategies for ROS homeostasis

The adaptive regulation of ROS balance is a complex and intricate process involving a variety of biological mechanisms and signaling pathways. In addition to oxidants and antioxidants, it also includes factors such as mitochondrial dynamics regulators, autophagy and mitophagy regulators, mitochondrial DNA repair agents, calcium homeostasis regulators, nutrient sensing pathway regulators, and oxygen adaptation mechanisms. Mitochondrial dynamics, including fusion and fission, respond to changes in the intracellular and extracellular environment by regulating mitochondrial size, number, and localization, thereby affecting both ROS production/clearance and organismal adaptive capacity. For example, Dynamin-related protein 1 inhibitors can prevent mitochondrial fission and reduce ROS production, thereby maintaining oxygen balance [519]. Autophagy and mitophagy maintain oxygen balance by degrading dysfunctional mitochondria and preventing excessive accumulation of ROS. Moreover, under hypoxic conditions, the activation of autophagy and mitophagy can reduce ROS production and protect cells from oxidative damage [255]. Urolithin A, a natural compound, can promote mitophagy and improve mitochondrial function and reduce oxidative stress in aging-related diseases [520]. Mutations and damage to mitochondrial DNA can affect the function of oxidative phosphorylation complexes, leading to increased ROS generation. Disruption of this adaptive ROS homeostasis further induces pathological manifestations. Idebenone, a mitochondrial electron transport chain regulator with antioxidant properties, has been used to treat mitochondrial diseases by reducing ROS levels and improving mitochondrial function [521]. Another typical example is Mitoquinone methanesulfonate, a mitochondria-targeted antioxidant that has been shown to reduce mtDNA oxidation and protect cells from oxidative stress [522, 523]. Disruption of calcium homeostasis can trigger mitochondrial dysfunction and increased ROS production. Diltiazem, a calcium channel blocker, has been shown to protect mitochondrial function and reduce oxidative stress in cardiovascular diseases [524, 525]. Nutrient sensing pathways, such as the mTOR pathway, affect ROS production and clearance by regulating cellular metabolism and growth [526]. ROS are closely regulated by the ambient oxygen environment; thus, therapies based on oxygen adaptation not only address hypoxemia but also influence ROS homeostasis. This indicates that the therapeutic goal should shift from merely suppressing ROS levels to enhancing the body's adaptive capacity to fluctuating oxygen and ROS conditions. Hypoxia and high-altitude adaptation promote the expression of genes related to oxygen homeostasis and antioxidant defense by upregulating HIFs. Dexamethasone, a glucocorticoid, treats altitude sickness by reducing inflammation and oxidative stress [527]. Another example is Erythropoietin, a hormone that promotes red blood cell production, which has been shown to protect cells from oxidative stress under low oxygen conditions [528, 529]. Exposure to hyperoxia triggers compensatory mechanisms to counteract excessive ROS production by activating antioxidant defense pathways and regulating mitochondrial function. For example, NAC treats hyperoxia-induced lung injury by reducing ROS levels and improving mitochondrial function [530, 531]. Based on the organism's adaptive regulatory mechanisms to oxygen environments, several oxygen adaptation-based therapies have been developed in clinical practice. Clinically, various oxygen therapies—including hyperbaric and high-flow oxygen treatments—not only improve tissue oxygenation but may also reprogram redox dynamics, influence antioxidant defenses, and indirectly modulate ROS signaling pathways. Building upon this, ROS-adaptive therapy emphasizes the importance of redox resilience, aiming to fine-tune the cellular response to oxidative fluctuations through mechanisms such as intermittent hypoxia preconditioning, NRF2 pathway activation, and modulation of redox-sensitive transcription factors. Rather than eliminating ROS entirely, these strategies seek to restore a dynamic balance, offering a physiologically aligned and sustainable approach to oxidative stress-related diseases, including chronic lung disorders [532] and cancer [499].

In summary, the adaptive regulation of ROS homeostasis involves a complex network of mechanisms and signaling pathways. Drugs targeting mitochondrial dynamics, autophagy, mtDNA repair, calcium homeostasis, and oxygen adaptation strategies play a key role in maintaining oxygen balance and offer promising therapeutic approaches for a variety of diseases.

## Perspectives and conclusions

As discussed above, intracellular ROS homeostasis is a fundamental aspect of cell physiology. It ensures that ROS levels are strictly regulated to meet metabolic demands while avoiding toxicity [533]. Disruption of ROS homeostasis can have profound impacts on cell survival, function, and adaptation [101, 534]. Under hypoxic conditions, intracellular molecules including HIFs actively coordinate the body's adaptive responses to ROS [535]. Conversely, excessive ROS can lead to oxidative stress, highlighting the delicate balance required for optimal cell function. Therefore, understanding the mechanisms that control ROS homeostasis is crucial for elucidating its role in health and disease [536].

It should be noted that although ROS play a central role in regulating oxygen homeostasis [537, 538], it was traditionally considered as harmful byproducts of aerobic metabolism but not key signaling molecules that regulate cellular processes until recent years [538, 539]. Under physiological conditions, ROS are produced in a controlled manner by mitochondria, NOXs, and other enzyme systems, and are strictly regulated by antioxidant defenses [540–542]. ROS levels fluctuate with changes in oxygen availability, creating a dynamic interplay between oxygen and ROS [543, 544]. The dual role of ROS as signaling molecules and potential toxins emphasizes their importance in maintaining oxygen homeostasis and their involvement in disease pathogenesis when dysregulated [545].

Meanwhile, the roles of ROS homeostasis should not be neglected in the regulation of various cellular processes, including cell metabolism, cell death, and stem cell function and differentiation [384]. In terms of metabolism, ROS affect numerous metabolic processes such as glycolysis, lipid oxidation, amino acid utilization, and nucleotide synthesis, shaping the intricate metabolic landscape of cells [154, 156, 167, 546]. For instance, ROS can activate AMPK, inhibit the mammalian target of rapamycin (mTOR), and promote catabolic processes under stress conditions [547, 548]. In the context of cell death, ROS are key mediators of apoptosis, necrosis, and ferroptosis, with their levels determining cell survival or death [214, 549]. Additionally, ROS play a pivotal role in stem cell biology by regulating both self-renewal and differentiation. Typically, low levels of ROS are associated with the maintenance of stem cell and self-renewal capacity, whereas elevated ROS levels can drive differentiation or induce cellular senescence. These findings underscore the multifaceted role of ROS as key modulators of cell fate decisions [16, 550].

The oxygen balance based on ROS has unimaginably widespread impacts at the microscopic level of individual cells, meaning that the interaction between oxygen homeostasis and ROS homeostasis spans the entire lifespan and is involved in the pathological processes of a wide range of diseases [551–554]. During development, oxygen gradients and ROS signaling are crucial for embryonic tissue patterning and organogenesis. In adulthood, ROS contribute to immune responses, tissue repair, and adaptation to environmental challenges. However, as age increases, the balance between ROS production and antioxidant defenses deteriorates, leading to chronic oxidative stress across organ systems. The pathophysiological processes universally converge on progressive depletion of endogenous antioxidants (e.g., SOD) and ROS-mediated amplification of macromolecular damage and cellular dysfunction [100, 555]. Moreover, in aging-related research, ROS have been star molecules, stemming from their continuous impact on tissue aging. Therefore, understanding the spatiotemporal dynamics of ROS in physiological and pathological settings is crucial for developing targeted intervention strategies.

Given the central role of ROS homeostasis in health and disease, targeting ROS and their mediated ROS balancing pathways holds great therapeutic potential [556, 557]. Strategies to modulate ROS levels include the use of antioxidants, ROS scavengers, and inhibitors of ROS-generating enzymes. However, the dual role of ROS as signaling molecules and toxins requires a delicate approach, as indiscriminate inhibition of ROS may disrupt physiological signaling [14, 539, 558]. Alternatively, enhancing antioxidant defenses by activating NRF2 or using mitochondria-targeted antioxidants provides a more selective strategy [222]. In the context of ROS homeostasis, agents such as HIF stabilizers or inhibitors are often crucial for the adaptive survival of tissue cells under hypoxic conditions [559]. Other interventions, including oxygen delivery systems and hyperbaric oxygen therapy, are currently being explored across a range of conditions, from cancer to ischemic diseases. These approaches largely focus on harnessing or enhancing the body’s adaptive responses to oxygen availability [560, 561]. It has to be mentioned that the importance of ROS in life processes means that the exploration of new regulatory targets based on ROS homeostasis has never stopped. The discovery of HIF has brought enough surprises to the research community, but it is believed that there are still other targets to be excavated, or more precise biological regulatory sites [562, 563]. The rapid development of high throughput strategies and CRISPR-Cas9 technologies is helping the research community to more precisely find the treasures among them.

Of course, novel therapeutic strategies based on ROS mechanisms are not limited to antioxidant strategies. In fact, ROS-modulating therapies share certain similarities with existing treatments such as chemotherapy and immunotherapy, that is, pro-oxidative stress strategies to further kill tumor cells and other cells, which has been recognized as a promising approach to improve treatment outcomes for decades [564, 565]. Therefore, exploring targeted pro-oxidative stress therapeutic strategies will be a promising direction. Some nanformulations are already making efforts in this regard, for example, targeted nanotherapy has attracted a great deal of attention and effort from scientists, and has made extensive progress in basic research [35, 566–568].

In addition, considering the body as an adaptable and sensate organism with the potential for strong survival adaptation, oxygen-sensing-based oxygen therapies or oxygen-adaptability therapies are also being gradually attempted. For example, oxygen therapy, an increasingly studied clinical intervention, leverages varying oxygen concentrations or pressures to treat a range of conditions, including COPD and oxygen toxicity. Its therapeutic essence lies in reprogramming the body’s perception of both the oxygen and ROS environments, thereby restoring cellular vitality and enhancing the potential for self-renewal and functional recovery [569–571]. On the other hand, for chronic diseases or from a physiological perspective, changing the body's continuous ROS-adaptability strategy may also be an effective attempt to resist aging and promote metabolic health [572, 573]. Although the mechanisms are not yet clear, they may involve mitochondrial energy metabolism, DNA damage repair, and so on. However, the essence of these therapies is to find an appropriate window for dealing with diseases and long-term anti-aging survival based on the body's rapid or slow oxygen adaptation.

In conclusion, the far-reaching impact of ROS and the oxygen-balancing process in the body will be a lasting topic in the future. It is necessary to have a deeper understanding of the role of ROS in oxygen balance, and to dynamically view the impact of ROS homeostasis on cells and the body. Regulation based on ROS homeostasis holds great potential for maintaining physiological homeostasis and treating diseases. Many emerging intervention strategies are already under investigation. However, future research should also prioritize elucidating the environment-specific roles of ROS homeostasis, identifying reliable biomarkers for patient stratification, and integrating precision medicine approaches to effectively harness these pathways for disease treatment.

## Data Availability

Not applicable.
